# The Genetic Basis of Scale-Loss Phenotype in the Rapid Radiation of *Takifugu* Fishes

**DOI:** 10.3390/genes10121027

**Published:** 2019-12-10

**Authors:** Dong In Kim, Wataru Kai, Sho Hosoya, Mana Sato, Aoi Nozawa, Miwa Kuroyanagi, Yuka Jo, Satoshi Tasumi, Hiroaki Suetake, Yuzuru Suzuki, Kiyoshi Kikuchi

**Affiliations:** Fisheries Laboratory, University of Tokyo, Maisaka, Shizuoka 431-0214, Japan; skstjdrhd2012@gmail.com (D.I.K.); Wataru.Kai@fluidigm.com (W.K.); ahosoya@mail.ecc.u-tokyo.ac.jp (S.H.); oracion229@yahoo.co.jp (M.S.); azure82_09skyblue@yahoo.co.jp (A.N.); miwayoshiura_080401@yahoo.co.jp (M.K.); ayuka@mail.ecc.u-tokyo.ac.jp (Y.J.); tasumi@fish.kagoshima-u.ac.jp (S.T.); suetake@fpu.ac.jp (H.S.); yuzuru.suzuki@xui.biglobe.ne.jp (Y.S.)

**Keywords:** QTL mapping, rapid adaptation, phenotypic divergence, pufferfish, scale morphology, convergence, developmental diversity

## Abstract

Rapid radiation associated with phenotypic divergence and convergence provides an opportunity to study the genetic mechanisms of evolution. Here we investigate the genus *Takifugu* that has undergone explosive radiation relatively recently and contains a subset of closely-related species with a scale-loss phenotype. By using observations during development and genetic mapping approaches, we show that the scale-loss phenotype of two *Takifugu* species, *T. pardalis* Temminck & Schlegel and *T. snyderi* Abe, is largely controlled by an overlapping genomic segment (QTL). A search for candidate genes underlying the scale-loss phenotype revealed that the QTL region contains no known genes responsible for the evolution of scale-loss phenotype in other fishes. These results suggest that the genes used for the scale-loss phenotypes in the two *Takifugu* are likely the same, but the genes used for the similar phenotype in *Takifugu* and distantly related fishes are not the same. Meanwhile, *Fgfrl1*, a gene predicted to function in a pathway known to regulate bone/scale development was identified in the QTL region. Since *Fgfr1a1*, another memebr of the Fgf signaling pathway, has been implicated in scale loss/scale shape in fish distantly related to *Takifugu*, our results suggest that the convergence of the scale-loss phenotype may be constrained by signaling modules with conserved roles in scale development.

## 1. Introduction

Rapid evolutionary radiation generates both phenotypic disparity and similarity. Classic examples of the rapid radiation include finch birds, silversword plants, anole lizards, threespine sticklebacks, and cichlid fishes [1,2,3,4,5,6]. Recent advances in genomics have enabled the study of the genetic basis of phenotypic evolution associated with rapid adaptation not only in the classic examples but also in many other groups of organisms [7,8]. Interestingly, this line of research also allows investigations into the predictability of evolutionary change [9,10,11,12,13]. However, more studies are needed for a better understanding of genetic architecture that promotes diversification, which would facilitate the stable prediction of evolutionary changes.

The scale is attractive for comparative studies of phenotypic evolution since it is widely distributed among vertebrates, and shows an impressive diversity in morphology [14,15,16]. For example, scale loss or reduction is a recurring feature in a number of teleost lineages, such as anguilliformes (eels), cypriniformes (carps), gasterosteiformes (sticklebacks), and siluriformes (catfish) [17,18,19,20,21,22,23]. Detailed studies on the three-spine stickleback (*Gasterosteus aculeatus* Linnaeus) have revealed that a specific allele of the ectodysplasin (*Eda*) gene, fixed in most freshwater populations, is associated with the convergent loss of lateral plates (modified scales) [17,24]. In addition, mutations in fibroblast growth factor receptor 1a1 gene (*Fgfr1a1*) are associated with the scale reduction of common carp *(Cyprinus carpio L.*) and zebrafish (*Danio rerio* Hamilton), whereas a mutation in the ectodysplasin receptor gene (*Edar*) leads to almost complete loss of scales in medaka (*Oryzias latipes* Temminck & Schlegel) [22,25].

**Figure 1:**
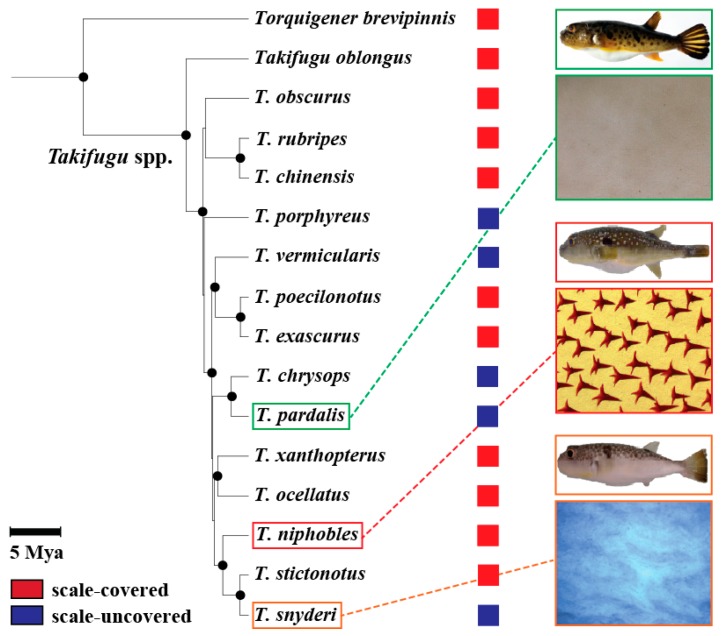
Phylogenetic relationship of 14 *Takifugu* and 1 other pufferfishes, and their scale phenotypes. The phylogeny is redraw using data from [29] where mitochondrial DNA data of 14 *Takifugu* and other related species have been analyzed using maximum likelihood method. Nodes supported by high bootstrap values (≥80%) are shown in black circles. Scale bar represents 5 million years before present. Also shown was a summary of previous results from [28] on the scale phenotypes for each species. The scale-covered (red) or -uncovered (blue) phenotypes are illustrated by colored squares on the right of each species. Examples of mineralized scales on the ventral surface of the body of three species, *T. pardalis*, *T. niphobles* and *T. snyderi*, were shown under the picture of each species.

Fugu (*Takifugu rubripes* Temminck & Schlegel) and its closely related species, being a prominent example of recent adaptive radiation in marine fishes, provide an opportunity for understanding the genetic basis of phenotypic evolution of the scale morphology for the reasons set forth below. Genus *Takifugu* underwent an explosive radiation in the last 2–5 million years, resulting in approximately 20 extant species, and the evolutionary history has been studied for 15 of them [26,27]. While most *Takifugu* species have spiny scales covering the integument (the scale-covered phenotype), at least five species show a loss or massive reduction in the number of scales, resulting in the “scale-loss” or “scale-uncovered” phenotype [28] (Figure 1). Interestingly, previous phylogenetic studies and phenotypic descriptions showed incongruences between phylogenies derived from mitochondrial DNA (mtDNA) data and morphological data [28,29] (Figure 1). These incongruences imply that the scale-uncovered phenotype evolved repeatedly in this genus, although a robust phylogeny using nuclear genes of the Takifugu fishes is currently lacking. In addition, *Takifugu* can produce fertile progeny when crossed with closely related species, which permits the genetic mapping of implicated genes [30,31,32]. 

Here, we aim to understand the genetic and developmental basis of morphological divergence/convergence associated with rapid radiation of *Takifugu* species in marine ecosystems. To this end, we focused on one species with the scale-covered phenotype (*T. niphobles* Jordan & Snyder) and two species with the scale-uncovered phenotype (*T. pardalis* and *T. snyderi*) and performed longitudinal observations of scale development in the three species. We then conducted quantitative trait locus (QTL) mapping using hybrid crosses of species exhibiting the scale-covered and scale-uncovered phenotypes (*T. niphobles* × *T. pardalis* and *T. niphobles* × *T. snyderi*).

## 2. Materials and Methods 

### 2.1. Developmental Pattern of Spiny Scales

To observe the developmental pattern of scales in *Takifugu* species, three closely-related species, one scale-covered (*T. niphobles*) and two scale-uncovered (*T. pardalis* and *T. snyderi*), were obtained by in vitro fertilization and sampled at 1, 2, 3, 5, 7, 14, 21, 28, 29, 56, 63, and 101 days post-hatch (dph). Live fish were stained up to 63 dph with 0.1% alizarin red S or 2% cochineal dye (Kiriya Chemical, Osaka, Japan) dissolved in half-strength seawater for 8 hours, washed in half-strength seawater for 10 min, and then euthanized in ice-cold seawater. For the 101 dph fish, the whole body was fixed in 10% buffered formalin for several weeks, and the ventral skin at the level of the pectoral fin was cut out. The dissected skin was placed into 0.1% alizarin red S in 1% KOH for 24 h and transferred into 1% KOH for three to six weeks to make the specimens transparent. Fluorescent images of scales of the three species were obtained using a stereoscopic microscope (MZ16F, Leica Microsystems, Wetzlar, Germany) or confocal microscope (FV1000, Olympus, Tokyo, Japan), and saved in JPEG format.

### 2.2. Hybrid Crosses 

*T. niphobles* (scale-covered) were captured from Washizu port (Hamamatsu, Shizuoka: 34°43’ N, 137°33’ E), while *T. pardalis* and *T. snyderi* (both scale-uncovered) were obtained from Sagara port (Omaezaki, Shizuoka: 34°41’ N, 138°12’ E). All three species were transferred to Fisheries Laboratory, University of Tokyo. Interspecific progeny were produced by in vitro fertilization using male *T. niphobles* and a female from either of the two scale-uncovered species (Figure S1). F_2_ progeny of *T. niphobles* × *T.* *pardalis* (NP-F_2_) were obtained by crossing the NP-F_1_s (F_1_ hybrids produced from a male *T. niphobles* and a female *T. pardalis*). From the NP-F_2_s, a total of 109 F_2_ fish at 104–122 dph and 358 fish at 149 dph were used for genome- and chromosome-wide mapping, respectively. In parallel, F_1_ hybrids between a male *T. niphobles* and a female *T. snyderi* (NS-F_1_) were also generated. Next, the backcross (NS-BC) was done by crossing a male F_1_ hybrid with a female *T. snyderi*. A total of 87 fish at 42 dph were used for genome-wide mapping, whereas 203 fish at 42 dph and 222 fish at 110 dph were used for chromosome-wide mapping. In addition, F_2_ progenies of *T. niphobles* × *T. snyderi* (NS-F_2_) were obtained through crossing the F_1_ hybrids. From these, a total of 196 fish at 109–110 dph were used for chromosome-wide mapping.

### 2.3. Phenotyping

The scale phenotype on the dissected skin samples was noted after alizarin red S staining was done, as described above. First, we evaluated scales as a binary trait where individuals with clearly stained scales and those without were considered scale-covered and scale-uncovered, respectively. Individuals with very faint staining were treated as missing data and excluded from the analysis based on the binary data. Next, we quantified the total scale number, the total area occupied by the scales, and the average area per scale (“size”) within a 10 × 10 mm area. To determine the total scale area, the 10 × 10 mm microscope image was converted into 100 × 100 pixels using Photoshop CS5 software (Adobe Inc., San Jose, USA), and the pixels on the scales were counted using the ImageJ software package [33]. Finally, the data obtained for the total area of the scales was divided by the total number of scales within 100 × 100 pixels to calculate the size of a scale.

### 2.4. Genotyping

Genomic DNA was extracted using Quick Gene DNA tissue kit S (FUJIFILM), from the caudal fin preserved in 600 uL of TNES-urea buffer (50 mM Tris-HCl (pH 7.5), 125 mM NaCl, 10 mM EDTA, 8 M urea, 1% SDS) [34] at room temperature. For QTL mapping, microsatellite markers derived from genetic maps of fugu [35,36] were used except NS-BC. The microsatellite markers and SNPs were used for NS-BC. The protocol for PCR reaction is described elsewhere [35]. Fragment analysis of the PCR products was done using either an LI-COR 4300 DNA analyzer (LI-COR Biosciences, Lincoln, USA) or an ABI3130 genetic analyzer (Life Technologies Corporation, Foster City, USA). 

SNP genotyping was done for NS-BC progeny by means of Ion AmpliSeq target amplicon sequencing following [37]. A primer pool consisting of 970 pairs of custom primers was designed on the Ion AmpliSeq designer system (ThermoFisher: https://www.ampliseq.com accessed on July 2015). The genomic position and sequences of the primers are listed in Table S1. In brief, 40 ng of genomic DNA was used for the first PCR with the primer pool using multiplex PCR buffer (Multiplex PCR Assay Kit ver.2, Takara Bio, Kyoto, Japan). Primer digestion was done using USER enzyme (New England Biolabs, Ipswich, USA). After magnetic beads purification with the 1.8× volume of Agencourt AMpure XP (Beckman Coulter), PCR products were end repaired, dA-tailed and ligated to the NEBNext adaptor using NEBNext Ultra Library prep reagents (New England Biolabs). Indexed libraries using P7/P5 adaptors were pooled and sequenced on the Illumina MiSeq with MiSeq reagent kit v2 (300 cycles, Illumina). The raw reads were quality-trimmed and mapped onto the fugu reference sequence (UCSC table browser assembly Oct. 2011 (FUGU5/fr3)) using BWA-mem with default parameter. Subsequently, genotype calling was done by jointly analyzing 87 individuals with grandparents and parents using GATK v3.6. gVCF files were first generated for each sample with a GATK-HaplotypeCaller and merged into a single file with the GATK-CombineGVCFs command. Then joint genotyping was called with GATK-GenotypeGVCFs. Effective loci where the segregation pattern of paternal alleles could be distinguished were visually selected on IGV [38] and used for QTL analysis. Loci genotyped less than 80% of the individuals were excluded. When more than one SNP was detected on a target locus, the SNP at the 5’-end were used.

### 2.5. Linkage Map Construction and QTL Mapping

Genome-wide linkage maps for NP-F_2_ and NS-BC were constructed following the method described in [39] using R/qtl [40]. The map distances between markers were calculated using Kosambi’s mapping function. Calculation of recombination fractions and LOD scores for all marker pairs was done using *est.rf* function. Each marker order was arranged by *ripple* function (see supplementary material for an example of the R script).

Genome- or chromosome-wide QTL analysis was done by means of the R/qtl package [40] with the step size of 1 cM. A permutation test (10,000 times) was performed to determine the LOD thresholds for significant (*p* < 0.05) and highly significant (*p* < 0.001) QTLs. The 95% credible interval (CI) of the QTL was determined by the *bayesint* function. Phenotypic variation explained (PVE, %) by each QTL was calculated by means of a drop-one-term analysis following multiple QTL model fitting via multiple imputations as described in the manual (see supplementary material for an example of the R script).

Genotype effects on phenotypes were statistically analyzed in the R statistical environment version 3.6.1 (R development core team, 2010) as follows. Phenotypic data evaluated as a binary trait were analyzed using general linear models (GLM) with binomial error distribution and logit link function. If there were significant differences among genotype categories, post hoc pair-wise comparisons were implemented using multcomp::glht [41]. Phenoypic data evaluated as quantitative traits were analyzed by using a non-parametric Kruskal–Wallis test for independent groups, followed by a non-parametric multiple comparison Dunn’s test.

### 2.6. Complementation Test

When each of two strains has a recessive Mendelian mutation that produces the same mutant phenotype with complete penetrance, complementation will occur only if the mutations are in different genes. The same logic would be applied when penetrance is not complete but high to some extent [17]. Therefore, complementation tests would provide a hint about whether or not the same gene is involved in the scale-loss phenotype in two closely related species of *Takifugu*. To conduct a complementation test for the scale phenotype, we obtained F_1_ progenies between *T. pardalis* × *T. snyderi*, in addition to the above mentioned F_1_ hybrids (*T. niphobles* × *T. pardalis* and *T. niphobles* × *T. snyderi*), and raised them till 57 dph. Scale phenotypes for these F_1_ hybrids were observed under the fluorescent stereoscopic microscope following cochineal dye staining, as described above. 

### 2.7. Candidate Gene Search

To identify candidate genes responsible for the scale-loss phenotype, we searched genes in fugu reference genome databases [42]. We then retrieved confidence limits to the QTL overlapping regions, and a list of genes residing in the homologous genomic region of fugu was obtained from the NCBI Entrez Gene database (https://www.ncbi.nlm.nih.gov/gene).

### 2.8. Candidate Gene Mapping

Since mutations in *Eda*, *Edar,* and *Fgfr1* are known to cause morphological evolution in fish scales [5,22,25,43], we obtained the genomic sequences of those genes and their flanking regions from fugu genome databases [42] to search for microsatellite loci anchored to these candidate genes. Primers for microsatellite markers were designed as previously described [36]. Genotyping and linkage mapping were performed by the method described above. 

### 2.9. Ethics and Data Availability

Experiments were approved by the IACUC (Institutional Animal Care and Use Committee) of the Graduate School of Agricultural and Life Sciences, University of Tokyo (P-170529004). All methods were carried out in accordance with the IACUC guidelines and regulations. The genotype and phenotype data from the above experiments are available in Dryad (doi.org/10.5061/dryad.6q573n5vd).

## 3. Results

### 3.1. Developmental Pattern of Spiny Scales 

**Figure 2:**
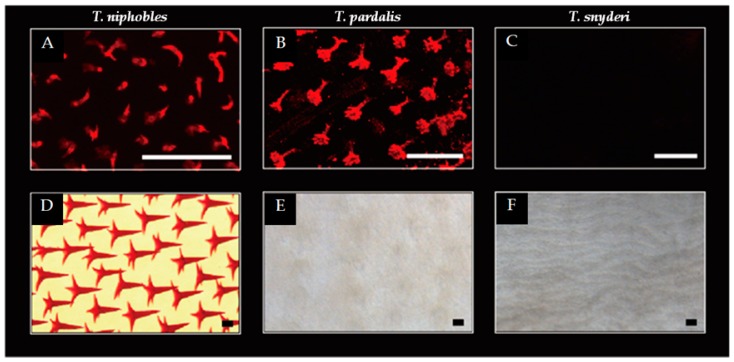
Scale developments in the *Takifugu* species. While scales were observed on the ventral surface of the body at the pectoral fin level in 9 dph (day post-hatch) larvae of *T. niphobles* (**A**) and *T. pardalis* (**B**), the scales were undetectable in *T. snyderi* (**C**). Well-developed spiny scales were observed in *T. niphobles* after 100 dph (**D**). The mineralized scales were almost undetectable in *T. pardalis* of 101 dph (**E**). The mineralized scales were undetectable in *T. synderi* over the course of our observation including 110 dph fish (**F**). Scale bars indicate 100 μM. The more detailed developmental process was described in Figure S2.

To learn what changes in scale formation could produce the scale-covered and scale-uncovered phenotypes, we followed the development of laboratory-reared fish obtained from within-species crosses of *T. niphobles*, *T. pardalis,* and *T. snyderi*. We observed remarkable differences among the three closely-related species. In *T. niphobles*, although no scales were seen on the fish at 1 dph (data not shown), scale mineralization was seen within 9 dph (Figure 2A and Figure S2A). Scale formation began from the anterior-ventral region of the abdomen (Figure S2A) and proceeded posteriorly to cover the entire surface of the abdomen by 28 dph (Figure S2C). The mineralized structure is in the shape of a hollow cone, and the tip of the cone is elongated to form a compact thorn (Figure 2D). This structure is similar to those described in other pufferfish species [16,28,44,45]. 

A similar pattern was observed in *T. pardalis* till 7-14 dph (Figure 2B and Figure S2G). However, the hollow cone structures were observed to shrink from 28 dph (Figure S2J), such that the mineralized scales were almost undetectable by 101 dph (Figure 2E and Figure S2K-L). In contrast, no scales were detected in *T. synderi* over the course of our observation (Figure 2C,F and Figure S2M-R). While the changes in scale development are likely to stem from the initiation of scale formation in *T. snyderi*, it seems that the changes are in the maintenance of the scales in *T. pardalis*. These results suggest that developmental changes associated with the scale-uncovered phenotype vary between *T. pardalis* and *T. snyderi.*

### 3.2. QTL Analysis

#### 3.2.1. Linkage Map

For genome-wide QTL interval mapping, we first constructed linkage maps for the hybrid crosses between the scale-covered and uncovered phenotypes (*T. niphobles* × *T. pardalis* and *T. niphobles* × *T. snyderi*). The linkage map of NP-F_2_ consists of 116 individuals and 125 microsatellite markers distributed across all 22 chromosomes of fugu (Table S2). It forms 22 linkage groups (LGs) spanning 2128.4 cM (Figure S3, Tables S3 and S4). The average number of markers per LG was 5.7, ranging from 4 to 10. The linkage map of NS-BC was constructed using SNP markers extracted from the amplicon sequences of 87 fish sampled at 42 dph (Table S1). Two hundred thirty-two informative markers covering the whole genome of fugu were selected, and eight microsatellite markers were used additionally. The map comprised 24 LGs with a total length of 622.4 cM (Figure S4, Tables S5 and S6). The average distribution of markers per LG was 10.0, with a range of 2–26 across 24 LGs. Among them, there were two pairs of LGs consisted of markers derived from the same fugu chromosome (Chr 13 and Chr 21, respectively). Therefore, these LGs were named LG13_1 and LG13_2, and LG21_1 and LG21_2, respectively. Marker orders in both maps were in perfect agreement with the previously published genetic map of fugu [36]. 

#### 3.2.2. Phenotypic Values

Scale phenotypes were collected when differences among the three species were apparent. These phenotypes were evaluated either as a binary trait (scale-covered or uncovered), or various quantitative traits (the number, the total area, and size in a 10 × 10 mm region). Among the NP-F_2_ progeny, the scale-covered phenotype dominated and segregated approximately in a 3:1 to 4:1 ratio (scale-covered:scale-uncovered). Scale-covered phenotype also dominated in NS hybrids. The two phenotypes segregated as 1:1 to 3:1 in NS-BC and 7:1 in NS-F_2_. The phenotypes of the crosses are summarized in Table S7. It should be noted that all the progeny categorized as “scale-uncovered” by visual inspection had spiny scales with zero pixels after quantification. 

#### 3.2.3. Genetic Basis of Scale Loss in *T. Pardalis*

**Figure 3:**
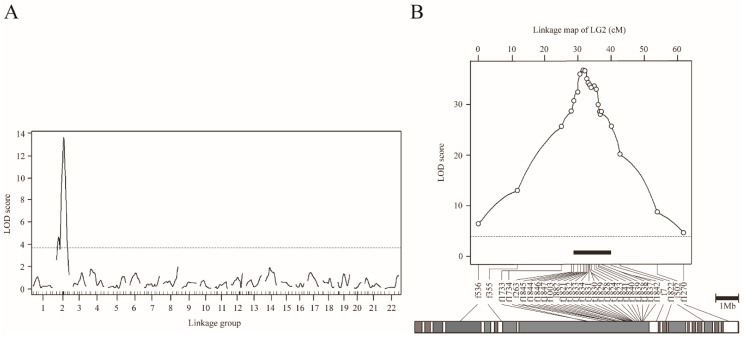
QTL mapping for scale reductions in NP-F_2_ progenies using the binary phenotype data. (A) Genome-wide mapping for 104–122 dph fish plotted over linkage groups. LOD score is on the *y*-axis, and linkage groups are on the *x*-axis. The numbering of the linkage group was based on homology to fugu chromosomes. Map position of markers is indicated by tick marks on the *x*-axis. LOD curves and significant levels are indicated by solid and dashed lines, respectively. The significance level (*p* = 0.05) of the LOD score determined by means of 10,000 permutations was 3.74 (dotted line). (**B**) Chromosome-wide QTL mapping for 149 dph fish plotted against linkage group 2. The LOD threshold for the chromosome-wide highly significant level (*p* = 0.001) determined by means of 10,000 permutations was 4.11 (dotted line). The black bar indicates the 95% credible interval for the QTL. The gray bar under the LOD plot describes the physical map of fugu chromosome 2.

In order to understand the genetic basis of the scale-uncovered phenotype in *T. pardalis*, we conducted genome-wide interval QTL mapping using NP-F_2_ progeny collected at 104-122 dph. By treating the scale as a binary trait, we successfully detected a highly significant QTL on LG2 (LOD = 13.6, Figure 3A, Table 1A). The f882 locus was the nearest to the LOD peak, and the locus accounted for 43.6% of the phenotypic variance explained (PVE). Similar QTL was detected when using the quantified phenotypic data (Figure S5). 

To refine the QTL position, we performed chromosome-wide analysis using fish at 149 dph, by increasing the number of genetic markers on LG2 from 11 to 31, and the sample size from 109 to 358 (Table 1B and Figure S1). By means of binary mapping, the maximum LOD reached 37.0 at a position near f1821 locus (Figure 3B, Table 1B). This analysis narrowed the 95% CI of QTL down to a region spanning approximately 1.8 Mb, flanked by f1846 (30.7 cM) and f1832 (32.5 cM) (black bar in Figure 3B). Similar results were obtained by analyzing the quantified phenotype (Figure S6). In particular, the 95% CI for the QTL for scale area was narrowed to roughly 1.2 Mb by means of fine mapping. 

**Figure 4:**
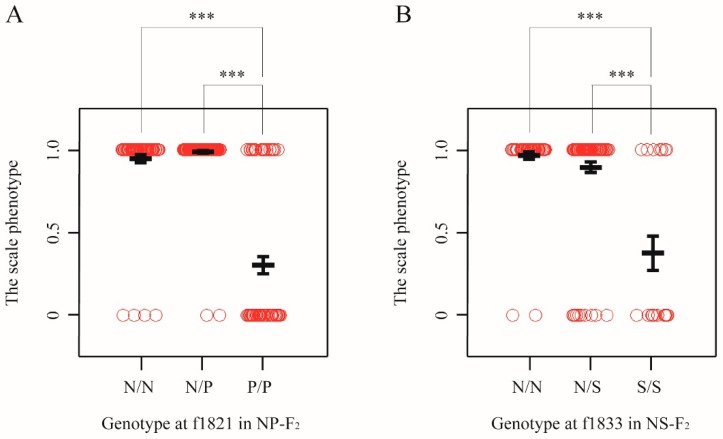
Effects of genotypes underlying binary traits. (**A**) Allele substitution effects in NP-F_2_ progeny at 149 dph for chromosome-wide mapping. The genotypes from marker locus nearest to the QTL peak are shown. The alleles derived from *T. niphobles* and *T. pardalis* are defined as N and P, respectively. Open circle shows the phenotypic value evaluated as binary data from each genotype. Black bold lines indicate the mean and error bars. Asterisks indicate significant difference between groups (adjusted *p* < 0.001) (**B**) Allele substitution effects in NS-F_2_ progeny at 109–110 dph for chromosome-wide mapping. The alleles derived from *T. niphobles* and *T. snyderi* are defined as N and S, respectively.

To estimate the allele substitution effects of the QTL, we examined the associations between phenotype and genotype on the marker locus nearest to the QTL peak of 104–122 dph and 149 dph fish (Table S8A). The result suggested that the QTL alleles derived from *T. pardalis* are largely recessive. For example, in 149 dph fish, individuals that were homozygous for the *T. pardali* (P/P) show a higher probability to exhibit the scale-uncovered phenotype than either of the homozygous individuals with the *T. niphobles* allele (N/N) or the heterozygous individuals (N/P) (adjusted *p* < 0.001, the Wald test; Figure 4A). Moreover, there are no significant differences in the phenotypic distributions in N/N genotype and N/P genotype (Figure 4A). When analyzing data from the 104–122 dph fish, the result is largely consistent with the recessive effect of the P allele (Table S8A). Similar results were obtained from the analysis using quantitative data (Figure S7).

#### 3.2.4. Genetic Basis of Scale Loss in *T. snyderi*

**Figure 5:**
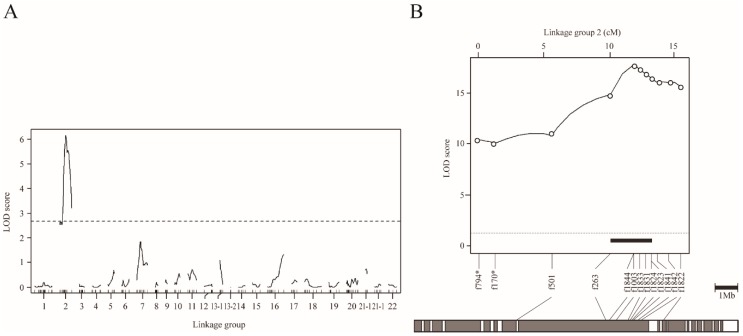
QTL mapping for scale reduction in NS-BC progenies using the binary phenotype data. (**A**) Genome-wide mapping for 42 dph fish plotted over linkage groups. LOD score is on the *y*-axis, and linkage groups are on the *x*-axis. The numbering of linkage group was based on homology to fugu chromosomes. Map position of markers is indicated by tick marks on the *x*-axis. LOD curves and significant levels are indicated by solid and dashed lines, respectively. The significance level of LOD score (*p* = 0.05) determined by means of 10,000 permutations was 2.55. (**B**) Chromosome-wide QTL mapping for 110 dph fish plotted against linkage group 2. The chromosome-wide highly significant level of LOD score (*p* = 0.05) determined by means of 10,000 permutations was 3.05. The black bar indicates the 95% credible interval for the QTL. The gray bar under the LOD plot describes the physical map of fugu chromosome 2.

To understand the genetic basis of the scale-uncovered phenotype in *T. snyderi*, we performed QTL analysis using NS-BC and NS-F_2_. Genome-wide mapping using 87 NS-BC progenies at 42 dph (binary model) detected a single highly significant QTL on LG2 (LOD = 6.1, Figure 5A, Table 1A), as for *T. pardalis*. The 7222201_17 locus nearest to the LOD peak accounted for 27.3% of the PVE (Table 1A). 

**Figure 6:**
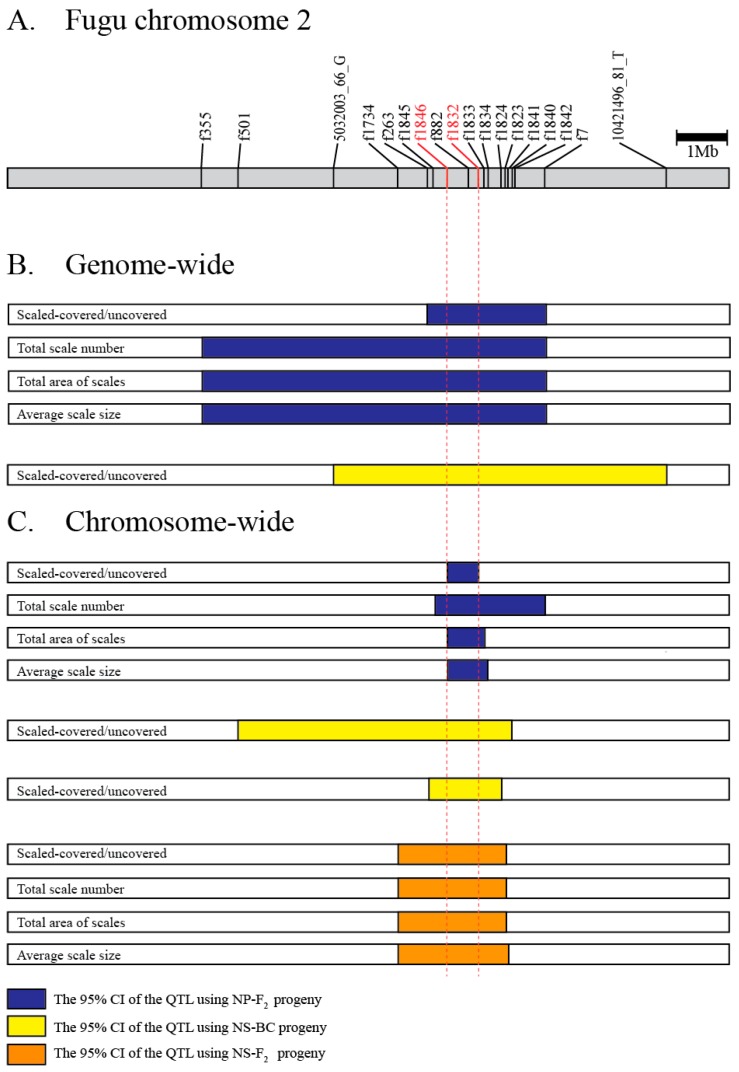
The overlapping QTL region. (**A**) Gray bar describes the physical map of fugu chromosome 2. Marker positions in fr3/FUGUv5 were indicated. (**B**) The 95% credible interval (CI) of QTL based on genome-wide mappings. (**C**) The 95% CI of QTL based on chromosome-wide mappings. The blue, yellow, and orange boxes indicate the 95% CI for NP-F_2_, NS-BC, and NS-F_2_, respectively. A pair of red dotted lines delimited the minimum overlapped region among the 15 intervals.

To refine the map position, we conducted a chromosome-wide analysis (binary model) with increasing sample size (from 87 to 203 for NC-BS at 42 dph) (Table 1B and Figure S8). The maximum LOD increased to 13.2 (near the f263 locus) and the 95% CI of the QTL contained approximately 4.6 Mb of the genome flanked by the loci f501 and f1840 (between positions 5.3 and 19.0 cM) (Figure S8, Table 1B). We then used another sample of 222 NS-BC fish at 110 dph, and obtained a slightly better resolution. The maximum LOD was 17.8 (f1003) (Figure 5B, Table 1B). The 95% CI of the QTL spanned approximately 3.2 Mb, flanked by f263 (10.1 cM) and f1824 (13.2 cM) (black bar in Figure 5B). The results of chromosome-wide mapping using 196 NS-F_2_ progeny at 109–110 dph were similar to those from NS-BC at 42 dph and 110 dph. (Figure 6 and Figure S9). Furthermore, the results based on quantitative data from NS-F_2_ were similar to those obtained from binary data (Figure S10). 

In general, the S alleles (*T. snyderi*) were recessive for the scale-uncovered phenotype in all experimental groups (NS-BC sampled at 42 dpf and 110 dph, and NS-F_2_ sampled at 109–110 dph) (Table S8B). For example, in NS-F_2_ progenies, while the phenotypic distributions in N/N and N/S genotypes at the f1833 locus are not significantly different each other, both are significantly different from that in S/S genotype (adjusted *p* < 1.0 × 10^−4^, the Wald test; Figure 4B). Quantitative data yielded similar results (Figure S11).

#### 3.2.5. Comparison of QTL Regions

Comparison of QTL regions between the hybrid crosses produced by crossing species with divergent scale phenotypes (*T. niphobles* × *T. pardalis* and *T. niphobles* × *T. snyderi*) revealed only one major QTL that exceeded the significance threshold for each species pair, and the genomic location of the QTL for both species pairs largely overlapped (Figure 6). In addition, the size and direction of QTL allele effects were also comparable (Table S8). These results suggest that either the same genes are involved or physically closely linked genes in the orthologous genomic region are involved in determining scale number, size, and coverage across species. 

### 3.3. Complementation Test

**Figure 7:**
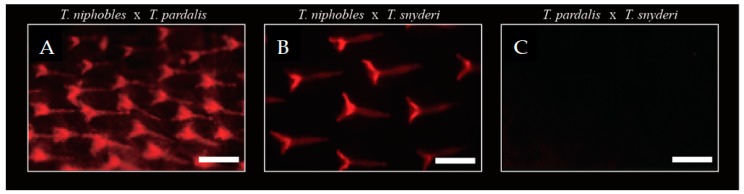
Complementation test. While scales were observed in F_1_ hybrids of *T. niphobles* × *T. paradalis* (57 dph) (**A**) and *T. niphobles* × *T. snyderi* (63 dph) (**B**), mineralized scales were undetectable in F_1_ hybrids at 57 dph of *T. paradalis* × *T. snyderi* (57 dph) (**C**). Scale bar = 200 μM.

QTL analyses suggested that the scale-uncovered phenotype in the two species, *T. pardalis* and *T. snyderi*, could be largely due to recessive mutations in the same gene. We tested this hypothesis by means of a complementation test. We obtained F_1_ hybrids by a diallel cross of the three species (*T. niphobles* × *T. pardalis* (NP), *T. niphobles* × *T. snyderi* (NS), and *T. pardalis* × *T. snyderi* (PS)), and observed their scale phenotypes (Figure 7). While the F_1_ hybrids of NP and NS showed a scale-covered phenotype (*n* = 3 for each cross), the F_1_ hybrid of PS exhibited a scale-uncovered phenotype (not complemented; *n* = 3). These results are consistent with the above-mentioned hypothesis. However, it should be noted that the QTL analyses also revealed that the scale trait is not a simple Mendelian trait. Therefore, more work is needed to understand the genetic basis of this trait. 

### 3.4. Candidate Gene Search

Our QTL analyses detected CI regions (95%) that were shared by all the phenotypes (binary and quantified) in all experimental crosses (Figure 6). However, it is unclear if the genes responsible for the binary and three quantitative phenotypes are the same since the ranges of 95% CI region varied even in the same mapped population. It is, of course, possible that the gene(s) responsible for the scale trait are in the overlapping regions and not the non-overlapping regions. Therefore, we searched for candidate gene(s) responsible for the scale-uncovered phenotypes in the overlapping segment present in all the analyses (indicated by vertical red dotted lines in Figure 6). Database mining revealed that this region corresponds to one physical sequence scaffold of approximately 0.74 Mb in the fugu genome (fr3/FUGUv5) in which 69 protein-coding genes have been annotated (Table S9). Since we were not able to find the genes that are known to cause morphological evolution in fish scales such as *Eda*, *Edar,* and *Fgfr1a* in the strict consensus overlapping segment of the QTL [5,22,25,43], we looked for a member of the signaling network implicated in scale development [15]. This search identified the gene encoding fibroblast growth factor receptor-like 1 (*Fgfrl1*). 

### 3.5. Candidate Gene Mapping

Although *Eda*, *Edar*, and *Fgfr1a* were not found in the fugu genome database corresponding to 95% CI of the QTL region on LG 2 that controls the scale-covered phenotypes (Table 2), it is possible that they could be translocated in the closely-related species used in this study. To exclude this possibility, we mapped the microsatellite markers anchored to these genes onto the linkage map using NP-F_2_ progeny. In addition, the genomic location of *Fgfr1b* genes was examined in the same way, since this gene is considered to be a teleost-specific paralog of *Fgfr1a* and is shared by many teleost species including pufferfishes. *Eda*, *Edar*, *Fgfr1a*, and *Fgfr1b* mapped to LGs 14, 1, 21, and 6, respectively, the same linkage group as in the fugu reference genome.

## 4. Discussion

Rapid radiation associated with phenotypic divergence and convergence has served as a model system for studying the mechanisms of evolution. Yet consistent features of the genetic and genomic basis underlying phenotypic diversification are not fully identified. To this end, we targeted the genus *Takifugu*, in which species exhibit diverged phenotypes for scale morphology associated with radial speciation. Using developmental observations and genetic mapping approaches, we found that the scale-uncovered phenotypes of *T. pardalis* and *T. snyderi* are controlled by an overlapping QTL (Figure 6). This is surprising since the phenotype in the two species had been suggested to evolve independently (Figure 1) and seemed to derive from slightly different developmental processes (the initiation/maintenance of scale formation) (Figure 2 and Figure S2). A search for candidate genes underlying the phenotype suggested that none of the known genes directly related to the scale reduction in other fishes (e.g., *Eda*) fall within the QTL region, ruling out their role as the major causative factor in the two *Takifugu* species. Our results, therefore, suggest that the major genes/mutations used for the scale-reduced phenotypes among *Takifugu* species are likely the same, but different from those of distantly related fishes (stickleback and *Phoxinellus alepidotus*) that show a similar phenotypic change. In addition, our search of the fugu genome annotation database for genes related to bone/scale development identified only one gene, *Fgfrl1*, from among those in the shared QTL region. Since *Fgf* signaling has been implicated in scale loss as well as scale shape variation in fish distantly related to *Takifugu*, it is possible that the convergence of the scale-reduced phenotype between distantly related species may be constrained by signaling modules used for scale development. 

Previous studies suggested the independence of the scale-reduced phenotype acquisition in *T. pardalis* and *T. snyderi* (Figure 1). Although our observation on the different patterns in scale development also corroborates with this view (Figure 2 and Figure S2), the genome-wide QTL analyses mapped their scale-uncovered phenotypes to a homologous region on fugu chromosome 2 (Chr 2) (Figure 6). These results suggest that the same genes are likely responsible for the loss of scales in the two lineages. The observed differences in scale development in the two species could be due to modifier loci other than the major QTL on Chr 2 since the QTL explains a maximum of 43.6% of the phenotypic variance (Table 1). In addition, the possibility cannot be excluded that different genes in the overlapping QTL region cause a similar phenotype, as this region spans a relatively large segment harboring around 69 genes (Table S9). It is also possible that different mutations in the same genes cause similar but different phenotypes, as clearly shown in the evolution of the naked cuticle of *Drosophila sechellia* [46].

It is interesting to speculate on the mechanisms involved if the same gene is used to manifest similar phenotypes in the two species. Independent mutations after the divergence of the two species constitute one possibility [47,48,49,50,51]. Alternatively, the mechanism could involve shared alleles via introgression from closely-related species, or incomplete lineage sorting of ancestral polymorphisms [6,52,53]. Although it is difficult to choose, based only on the results of our QTL analyses, between these two alternative mechanisms (independent or shared mutations) as the more likely explanation for the evolution of the scale-uncovered phenotype in *Takifugu* pufferfish, the possibility of the shared alleles is worthy of attention. In fact, natural hybridizations among *Takifugu* species have been reported [54,55,56]. Moreover, it has been suggested that past hybridization between differentiated/differentiating lineages may underlie the diversification of recently radiated groups by providing genetic variation and/or ancestral polymorphisms [2,57]. Thus, a search for the shared alleles specific to the two scale-uncovered *Takifugu* fish in the QTL region through genome sequencing is a reasonable step for future studies. Moreover, there are at least three additional species with the scale-uncovered phenotype among the 15 *Takifugu* species (Figure 1, [28]). Therefore, a genus-wide comparison of genomic sequences should be useful for understanding the historical events that have led to the divergence and convergent in scale morphology in this genus as in other taxa (e.g., [53,58]). 

The convergent evolution of traits in independent lineages inhabiting similar environments implies the action of natural selection [9]. It has been shown that repeated reduction of scales in stickleback is associated with the repeated adaptation of ancestral anadromous populations to freshwater environments [5,24]. However, the reduction of scales in *Takifugu* species is not likely to be associated with the freshwater environment. For example, *T. obscurus* and *T. ocellatus* have adapted to freshwater environments to spawn in the river, unlike most *Takifugu* species that spawn in the sea [59,60] (Nakamura et al. submitted). Yet, *T. obscurus* and *T. ocellatus* bear robust spine-like scales, just as the majority of *Takifugu* fish do (Figure 1). Moreover, scale-covered pufferfish appear to be more associated with brackish water habitats such as the river mouth than marine, when compared to the scale-uncovered pufferfish (Table S10, [28,61,62]). The evolutionary reasons underlying the evolution of the scale-uncovered phenotype in *Takifugu* remain to be determined.

However, caution should be taken for the above-mentioned discussion, since the phylogeny of *Takifugu* fishes shown by [27] (Figure 1) was based on mtDNA data only. Recent studies have shown that rapid radiation can result in the inconsistency in relationships between mtDNA and nuclear DNA, and even between nuclear segments among the species (e.g., [63]). A comparison of the whole genomes of the species belonging to *Takifugu* using high throughput sequencing will help to generate a robust phylogeny, which is required for a more reliable inference of the evolutionary transition between the scale-covered and scale-uncovered phenotype among *Takifugu* species.

Our search for candidate genes in the maximum and minimum intervals of QTL failed to identify any genes known to be causative for the scale-reduced phenotype in other fishes (*Eda*, *Edar,* and *Fgfr1a*) (Table 2 and Table S1) [5,22,25]. However, we found genes encoding Fgfrl1, a member of the receptor family of the Fgf signaling pathway. Fgf signaling has been implicated in the development of epithelial appendages of vertebrates, including scale development in teleosts [15]. For example, mutations in Fgf receptor genes were associated with independent scale loss in a domesticated lineage of carp as well as a related species of carp, *Phoxinellus alepidotus* [64]. In addition, it has recently been shown that Fgf signaling controls scale shape variation in a Lake Malawi cichlid (*Tropheops sp*.) [14]. Moreover, a pharmacological inhibition of Fgf signaling in zebrafish led to an immediate and complete arrest of scale initiation and outgrowth [15]. Fgfrl1 is a member of the fibroblast growth factor receptors, although Fgfrl1 lacks the cytoplasmic tyrosine kinase domain seen in all other family members. Fgfrl1 is essential for proper craniofacial skeletogenesis, particularly that involving the posterior gill cartilage in zebrafish [65]. Fgfrl1 is also indispensable for the development of the kidney and diaphragm [66,67,68]. Although no direct involvement of this gene in epithelial appendages has been reported (e.g., [65,69]), it is notable that Fgfrl1 has been shown to interact with many Fgf ligands, including Fgf8 and Fgf3, which were implicated in scale development in zebrafish and a pufferfish species, respectively [15,16]. The role of the candidate genes will be tested using the CRISPR/Cas9 technology, once the protocol for microinjection of nucleic acids into one-cell stage embryos is established in the *Takifugu* species.

## 5. Conclusions

The scale phenotypes among *Takifugu* provide a striking example of phenotypic evolution underpinned by the use of the same genomic segment associated with rapid radiation. Previous evidence of hybridization between these lineages and their recent divergence open up the possibility that alleles shared through introgression or incomplete lineage sorting have facilitated convergent adaptation between these species. Our study also indicates that the orthologous genomic segment is not used for the evolution of a similar phenotype in distantly related species such as threespine stickleback and *Phoxinellus alepidotus*. Similar examples of the convergence of other traits in other taxa have been compiled to yield insights into the predictability of evolution at the genetic level [9,12,51], and our results are largely consistent with their prediction that the probability of gene reuse for convergence is not high when comparing distantly related species. Nevertheless, caution is recommended for quantification using heterogeneous data, and the analysis restricting to a specific trait has been proposed as a reasonable alternative [51,70]. From this viewpoint, further studies on the loss/reduction of vertebrate scales would facilitate the stable estimation of the probability of gene (QTL) reuse in convergent evolution. In addition, further study of *Takifugu* fishes at the genome sequence level would help to understand genomic features that promote rapid radiation.

## Figures and Tables

**Table 1:** Summary of QTL analysis using binary and quantified phenotype for genome-wide mapping (**A**) and chromosome-wide mapping (**B**).

Cross	Scale Phenotype	Day Post Hatch (dph)	Sample Number	LG	Pos (cM)	Marker	95% CI (cM) ^a^	LOD	*P* value	PVE (%) ^b^	Add ^c^	Dom ^d^
A														
NP-F_2_	Covered/Uncovered	104–122	109	LG2	44.9	f882	12.6	13.6	2.8 × 10^−14^	43.6	−	−
	Total number of scales				45.0	f882	30.6	8.3	7.9 × 10^−10^	31.9	−5.8	3.6
	Total area of scales				41.0	f1003	30.6	9.0	5.8 × 10^−12^	37.8	−7.5	2.7
	The size of scales				41.0	f1003	30.6	8.7	1.5 × 10^−7^	25.6	−5.7	1.9
NS-BC	Covered/Uncovered	42	87		20.0	7222201_17	24.4	6.1	1.4 × 10^−7^	27.8	−	−
B														
NP-F_2_	Covered/Uncovered	149	358	LG2	32.5	f1821	1.8	37.0	0.00	37.9	−	−
	Total number of scales				35.0	f1823	11.3	9.57	1.4 × 10^−9^	10.8	−4.9	4.3
	Total area of scales				31.0	f1846	2.0	22.9	0.00	32.5	−11.0	7.5
	The size of scales				31.0	f1846	2.4	25.3	0.00	25.4	−9.5	5.9
NS-BC	Covered/Uncovered	42	203		15.1	f263	13.7	13.2	7.3 × 10^−15^	25.8	−	−
		110	222		11.9	f1003	3.2	17.8	0.00	30.8	−	−
NS-F_2_	Covered/Uncovered	109–110	196		38.0	f1833	14.1	8.4	6.3 × 10^−9^	17.5	−	−
	Total number of scales				43.0	f1831	14.1	5.1	1.0 × 10^−7^	15.1	−5.7	3.7
	Total area of scales				38.0	f1833	14.1	5.9	2.4 × 10^−7^	14.4	−5.6	3.3
	The size of scales				38.0	f1833	14.7	5.1	1.5 × 10^−4^	8.6	−4.0	3.0

^a^ 95% credible interval, ^b^ Phenotypic variant effect, ^c^ Additive effect, ^d^ Dominant effect.

**Table 2:** Candidate genes and their map position.

Gene Name	Position on fr3/FUGUv3			Marker Designing Region	Mapped Chromosomes
	Chr	Start (bp)	End (bp)		
*Eda*	14	5,873,023	5,884,580	<500 bp downstream	14
*Edar*	1	17,694,260	17,710,622	intron 1	1
*Fgfr1a*	21	5,867,819	5,885,613	<1 kb downstream	21
*Fgfr1b*	6	6,749,205	6,758,778	<5 kb downstream	6

## Supplementary Materials

The following are available online at https://www.mdpi.com/2073-4425/10/12/1027/s1, Figure S1: Mating scheme, Figure S2: Difference in morphology of spiny scales among *T. niphobles*, *T. pardalis*, and *T. snyderi*, Figure S3: Linkage map of NP-F_2_ progeny at 104–122 dph, Figure S4: Linkage map of NS-BC progeny at 42 dph, Figure S5: QTL mapping for reductions of spiny scales in NP-F_2_ progeny at 104–122 dph using quantized model, Figure S6: QTL mapping for the reduction of spiny scales in NP-F2 progeny at 149 dph using quantized model, Figure S7: Effects of genotypes on scale variations in NP-F_2_ progeny, Figure S8: QTL mapping for the reduction of spiny scales in NS-BC progeny at 42 dph using binary model, Figure S9: QTL mapping for the reduction of spiny scales in NS-F_2_ progeny at 109–110 dph using binary model, Figure S10: QTL mapping for the reduction of spiny scales in NS-F_2_ progeny at 109–110 dph using quantized model, Figure S11: Effects of genotypes on scale variation in NS-F_2_ progeny at 109–110 dph for chromosome-wide mapping, Table S1: List of the genomic position and primer sequences of each locus used for the linkage map construction of NS-BC family by means of target amplicon sequencing, Table S2: Information of microsatellite marker in NP-F_2_ progeny at 104–122 dph, Table S3: Summary of the linkage map in NP-F_2_ progeny at 104–122 dph, Table S4: Marker position of the linkage map in NP-F_2_ progeny at 104–122 dph, Table S5: Summary of the linkage map in NS-BC progeny at 42 dph, Table S6: Marker position of the linkage map in NS-BC progeny at 42 dph, Table S7: Summary of phenotypic values, Table S8: Effects of genotypes on spiny scale morph, Table S9: List of genes within the 95% credible interval flanking the major QTL on fugu chromosome 2, Table S10: The relationship between scale phenotypic and habitat in *Takifugu* species, Supplementary material for an example of the R script: Genetic mapping and QTL analysis.
