# Supplementary material for: The Genetic Basis of Scale-Loss Phenotype in the Rapid Radiation of Takifugu Fishes
Source: Genes (Basel). 2019 Dec 10;10(12):1027. doi: 10.3390/genes10121027 (PMC6947334; doi:10.3390/genes10121027)
Supplement: Supplementary file 1 [file genes-10-01027-s001.zip › supplementary files revised191206/SuppleFigure.docx]

**Supplementary material forThe genetic basis of scale-loss phenotype in the rapid radiation of Takifugu fishes.**

**
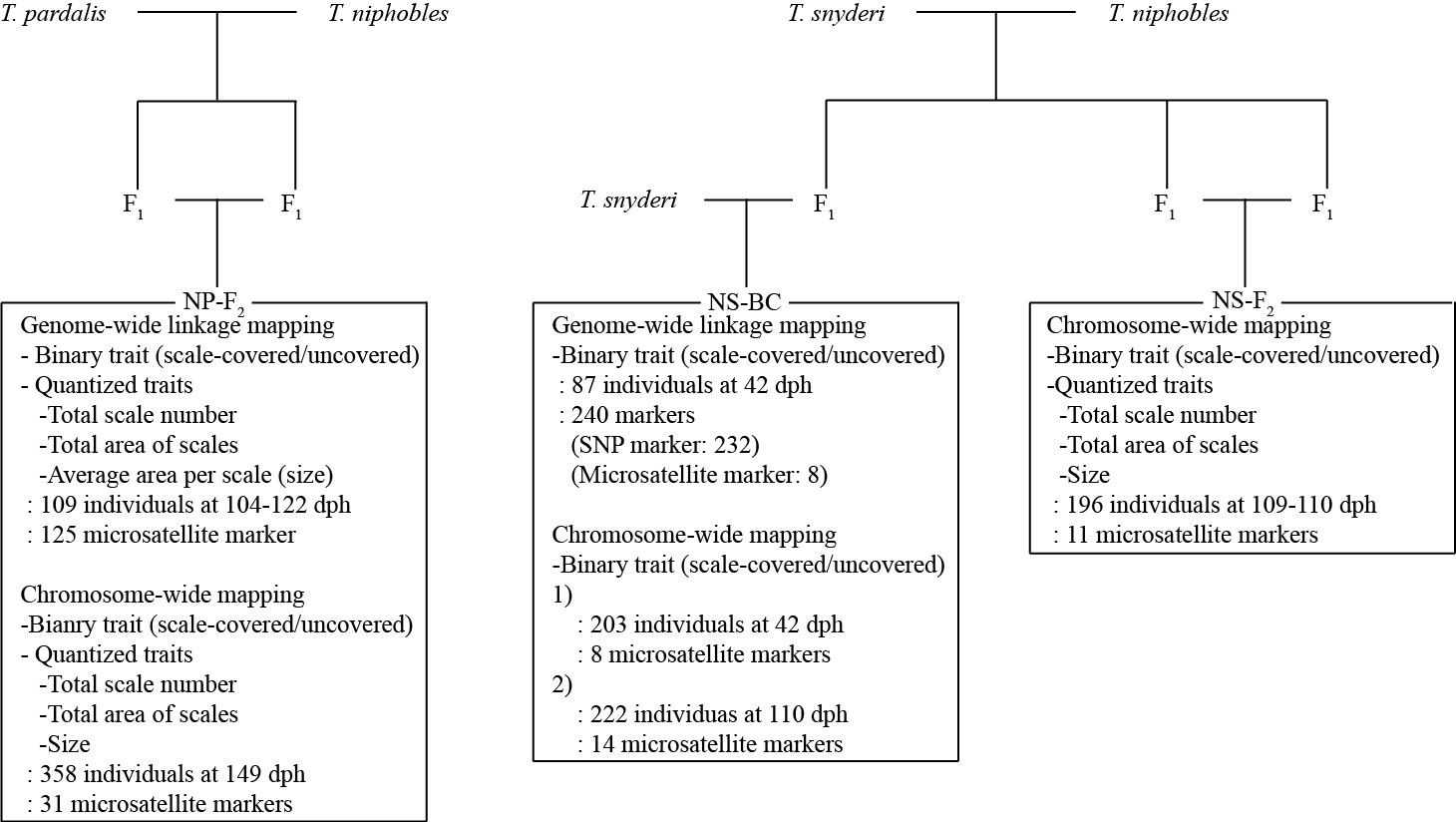
**

B

A

**Figure S1.** Mating scheme.

(A) A male *T. niphobles* and a female *T. pardalis* were captured and crossed in 2007 to obtain NP-F_1_ progeny. NP-F_2_ progeny was produced in 2009. (B) A male *T. niphobles* and a female *T. snyderi* were captured and crossed in 2014. Then a male F_1_ offspring was obtained and crossed with a female *T. snyderi* to produce NS-BC progeny in 2015. NS-F_2_ progeny was obtained in 2016 by crossing via brother-sister mating of the F_1_ progeny. The total number of progeny and marker loci used in each experiment are shown in the boxes.


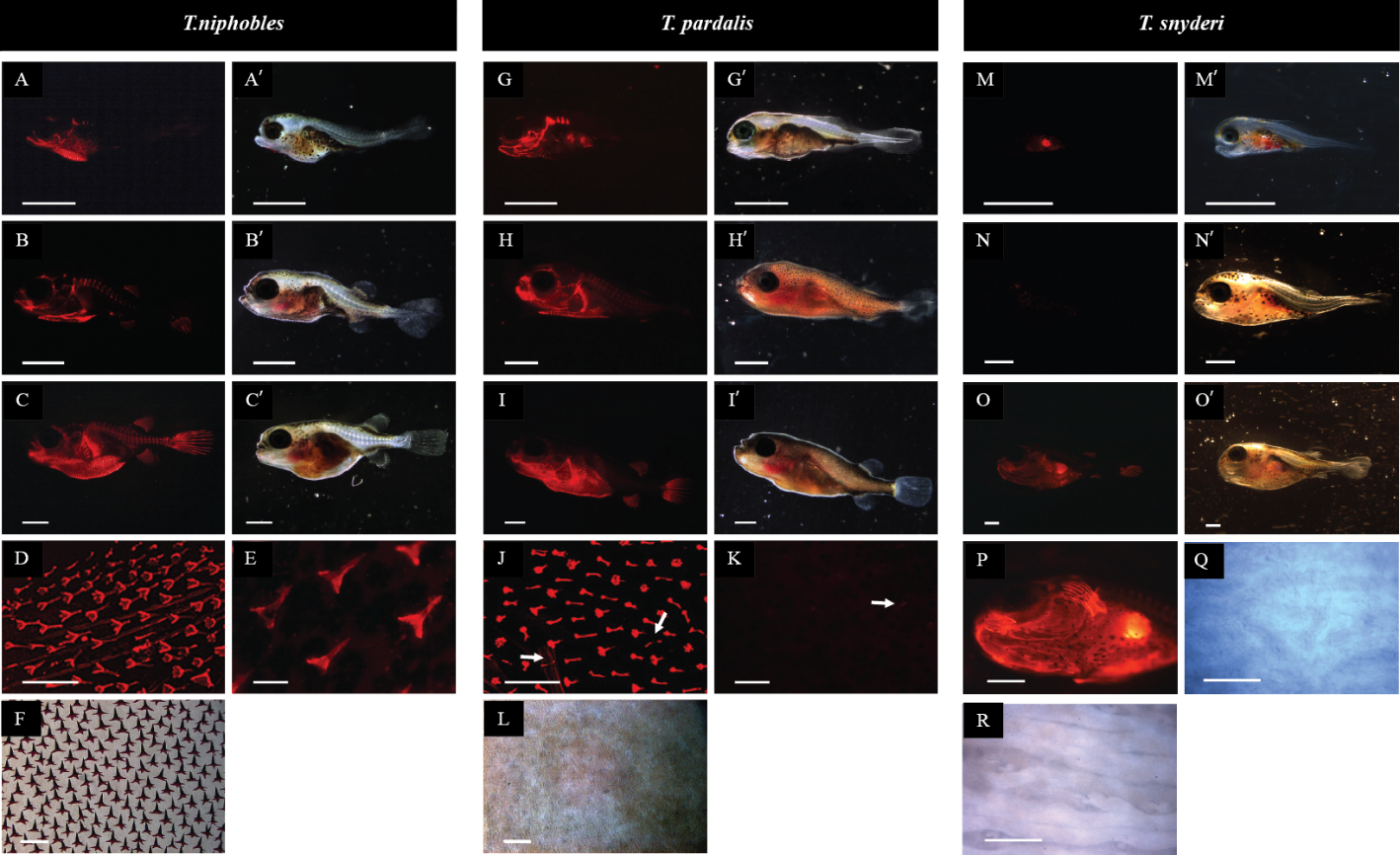


**Figure S2.** Development process of spiny scales in *T. niphobles*, *T. pardalis*, and *T. snyderi*. Fluorescence and corresponding bright-field images of *T. niphobles* at 7 (A and A'), 21(B and B'), 28 (C, C' and D), and 56 (E) days post hatch (dph), stained with 2% cochineal dye are shown. (F) *T. niphobles* at 100 dph stained with alizarin red S. *Takifugu pardalis* at 7 (G and G'), 21 (H and H'), 28 (I, I' and J), 63 (K), and 101 (L) dph. Atrophy of the base of spiny scale (the hollow cone) was observed by 28 dph (arrows in J) and the scales became undetectable by 63 dph (an arrow in K). *Takifugu snyderi* at 7 (M and M'), 21 (N and N'), 29 (O, O' and P) and 63 (Q) and 101 (R) dph. Spine-like scales in *T. snyderi* were not observed. Scale bars = 1 mm in A-C, F-I, L, and M- R. 200 μm in D, E, J and K.

**
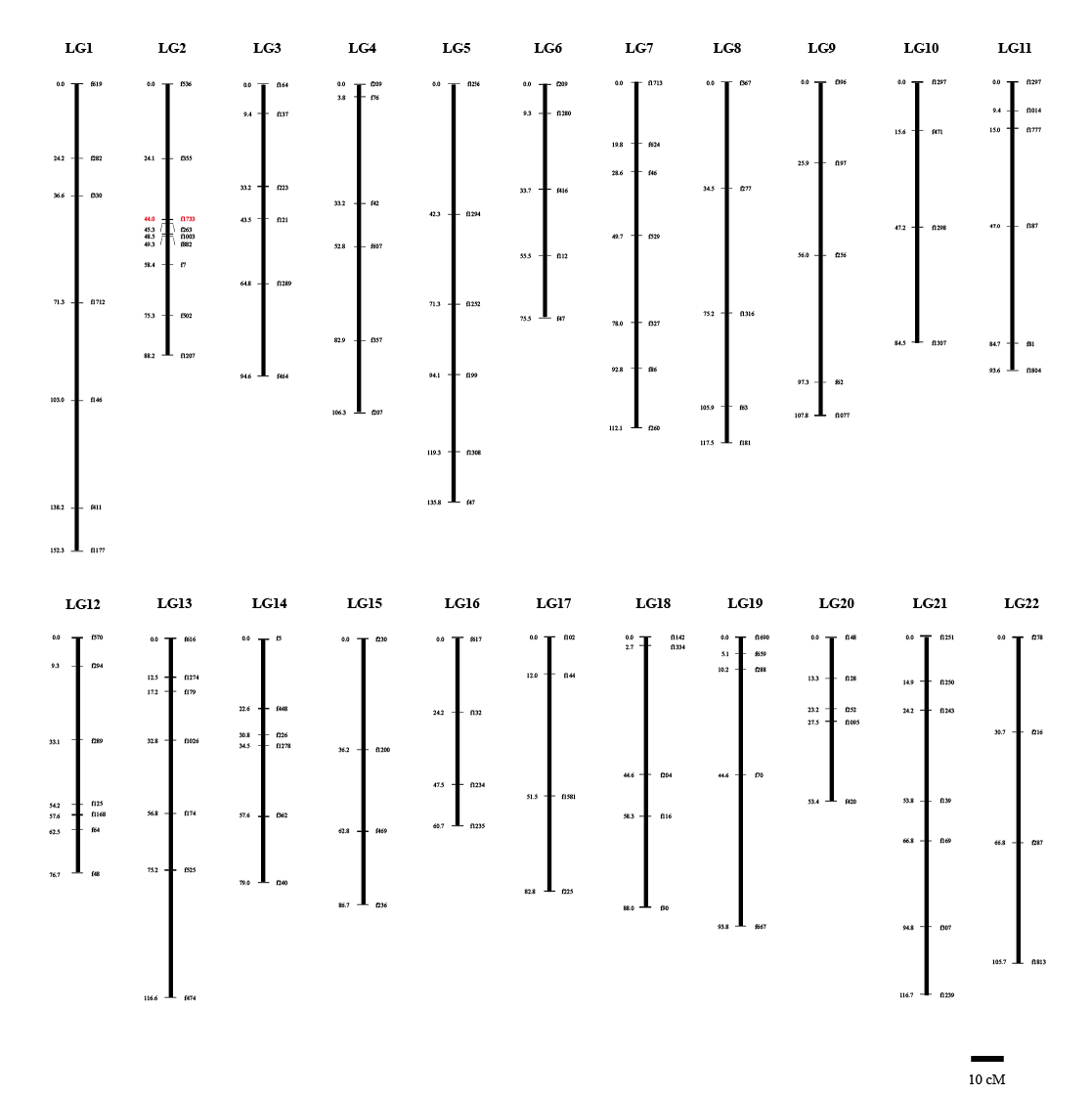
**

**Figure S3.** Linkage map of NP-F_2_ progeny at 104-122 dph. Vertical black bars represent linkage groups (LGs) for each chromosome. Map distances are shown in centimorgans (cM, Kosambi mapping function). The map consists of 22 LGs. Total genetic length is 2,128.3 cM. Two or more markers clustered within the range of 0.1 cM are indicated by red. Detailed marker information is provided in Table S4.

**
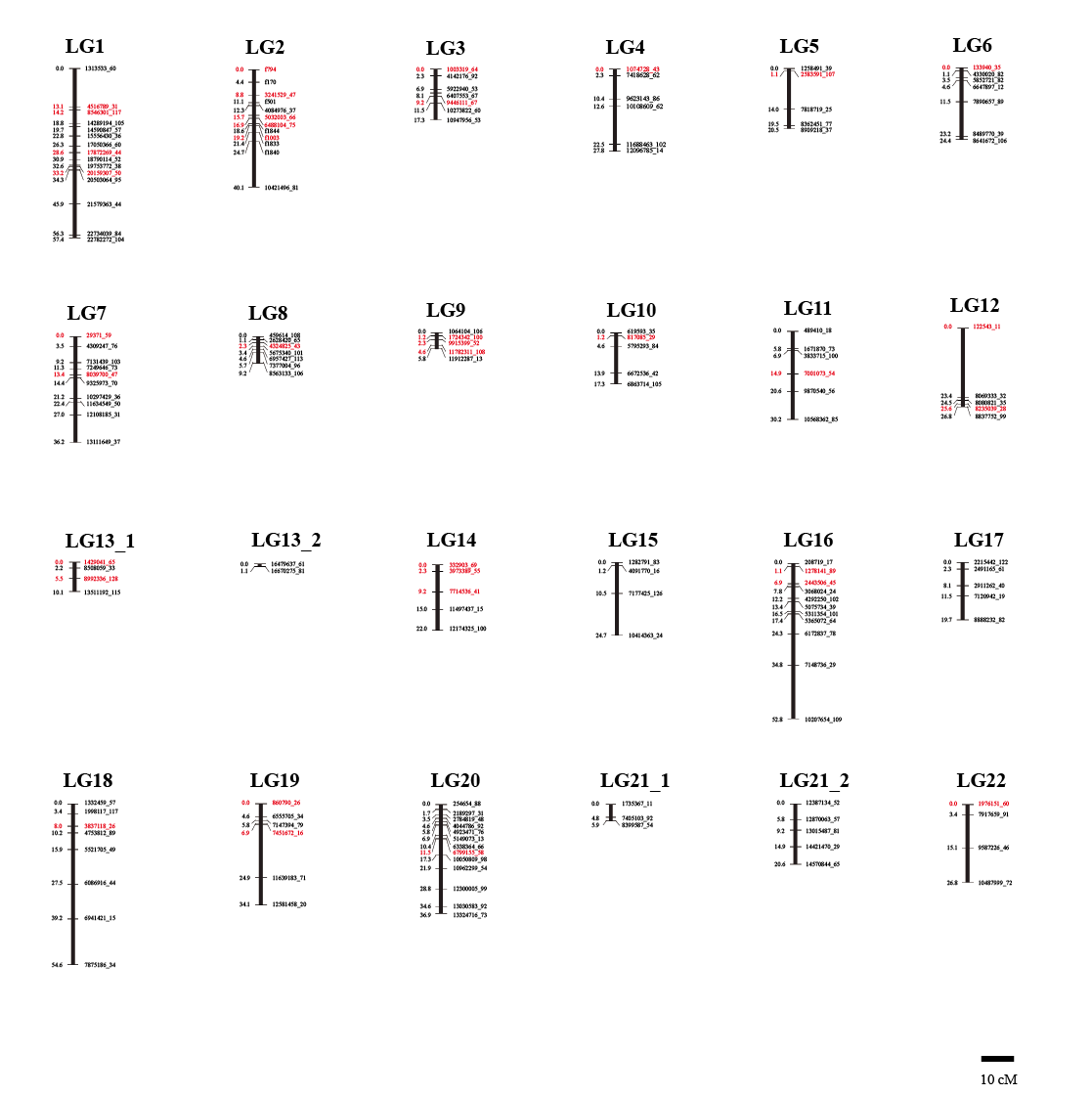
**

**Figure S4.**  Linkage map of NS-BC progeny at 42 dph. Vertical black bars represent linkage groups (LGs) for each chromosome. Map distances are shown in centimorgans (cM, Kosambi mapping function). The map consists of 24 LGs. Total genetic length is 622.4 cM. Two or more markers clustered within the range of 0.1 cM are indicated in red. Detailed marker information is provided in Table S6


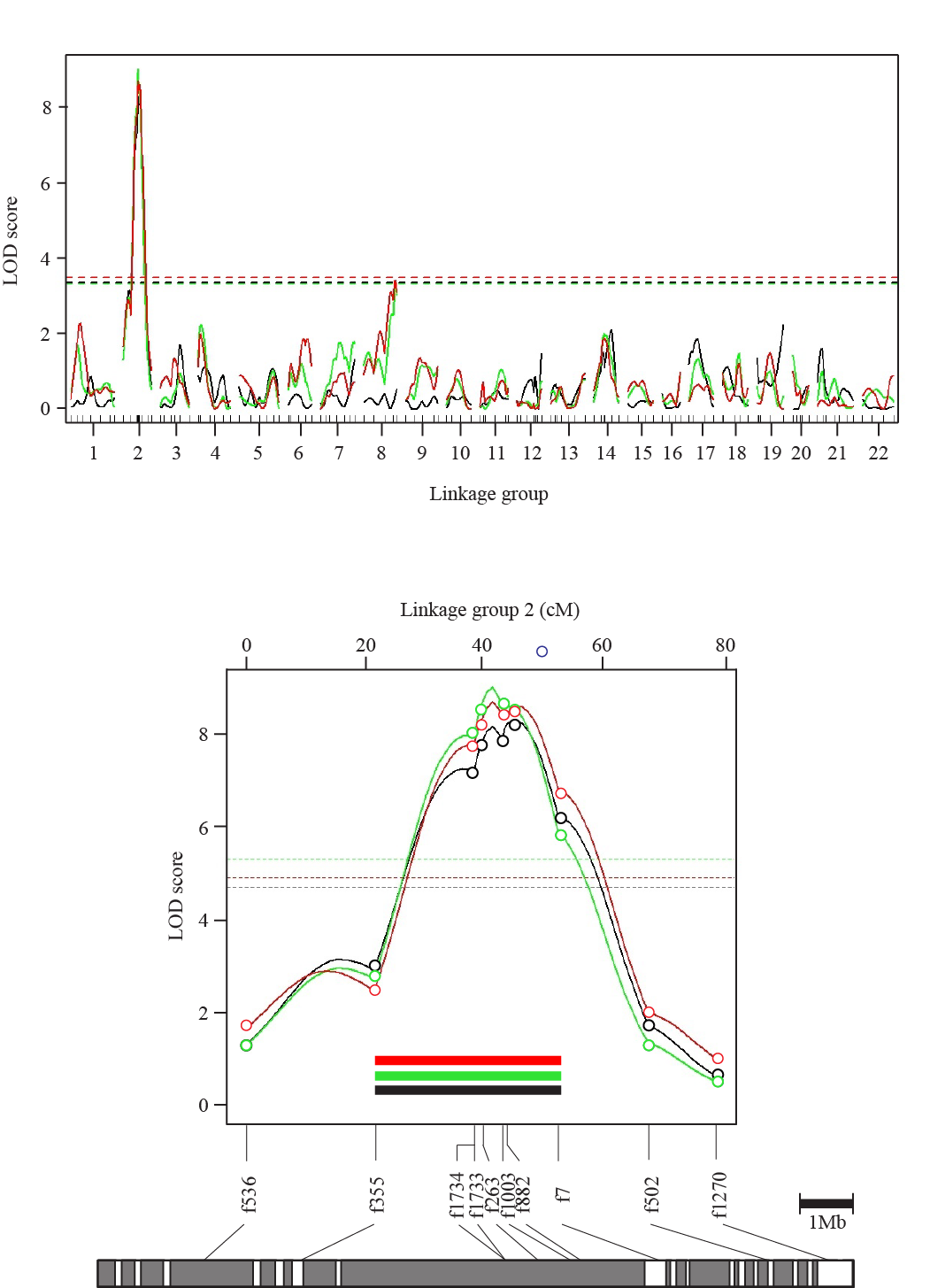


(A)

(B)

**Figure S5.**  QTL mapping for scale reduction in NP-F_2_ progeny at 104-122 dph using quantified phenotype. (A) Genome-wide mapping plotted over 22 linkage groups. LOD score is on the y-axis, and linkage groups are on the x-axis. Numbering of linkage group was based on homology to fugu chromosomes. Map position of markers is indicated by tick marks on the x-axis. LOD curves for the three traits (the total number, total area, and size of scale) are shown by black, blue, and red lines, respectively. The significance level calculated using a permutation analysis (n = 1,000) was LOD = 3.42, 3.38, and 3.46, respectively (*P* = 0.05). (B) Chromosome-wide QTL mapping plotted against linkage group 2. LOD curves and highly significant levels are shown by solid and dotted lines, respectively. The genome-wide highly significant level calculated using a permutation analysis (n = 10,000) was LOD = 4.84, 4.48, and 4.63, respectively. The 95% credible intervals for the three traits are indicated by black, blue, and red bars, respectively, under the LOD curve. The gray bar under the LOD plot depicts the physical map of fugu chromosome 2.


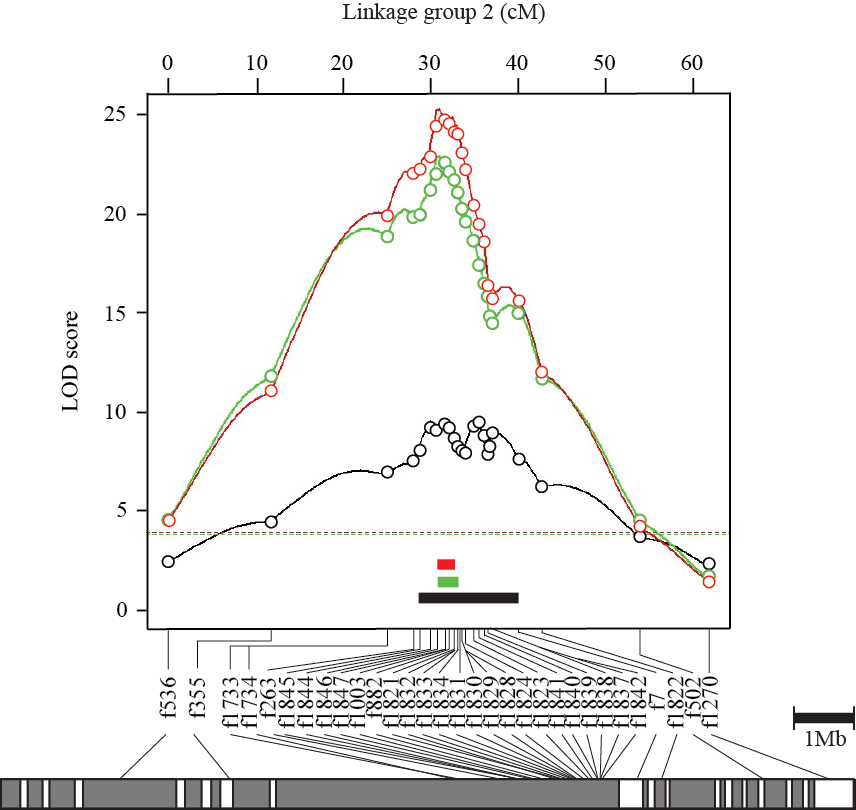


**Figure S6.** QTL mapping for the reduction of scales in NP-F_2_ progeny at 149 dph using quantified phenotype. Chromosome-wide QTL mapping plotted against linkage group 2. LOD score is on the y-axis, and map positions of markers on linkage group 2 are indicated under the x-axis. LOD curves for the three traits (the total number, total area, and size of scale) are shown by black, blue, and red lines, respectively. The chromosome-wide highly significant level calculated using a permutation analysis (n = 10,000) was LOD = 4.07, 4.23, and 4.17, respectively (*P*= 0.001). The 95% credible intervals for the three traits are indicated by black, blue, and red bars, respectively, under the LOD curve. Open circles show LOD scores of each marker. The gray bar under the LOD plot depicts the physical map of fugu chromosome 2.

**
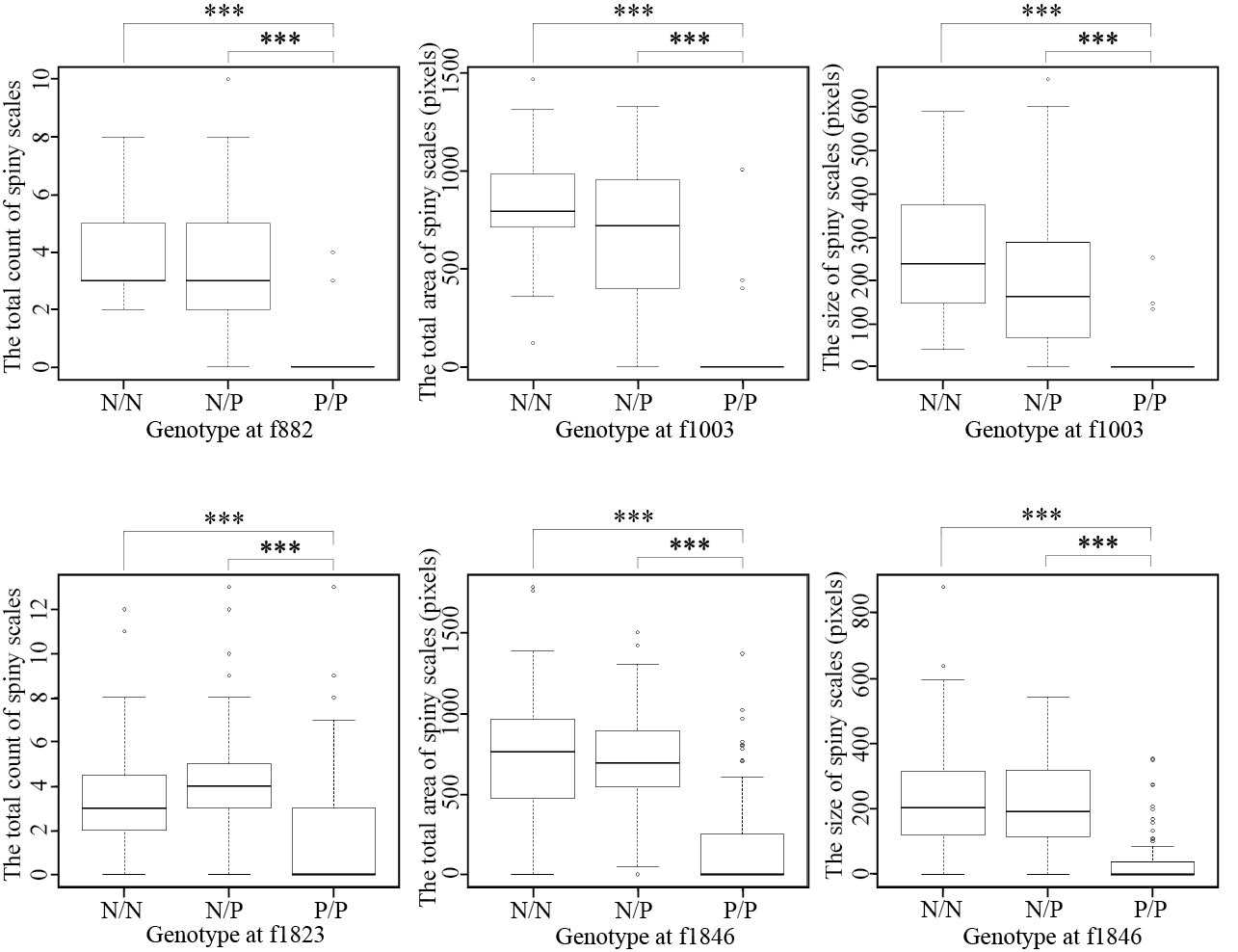
**

(B)

(A)

**Figure S7.** Effects of genotypes on scale variations in NP-F_2_ progeny. The genotypes from maker locus nearest to the QTL peak were shown. The allele derived from *T. niphobles* and *T. pardalis* are defined as N and P, respectively. Boxplot shows distributions of three quantified traits, the total count, total area of scales, and the average area per scale from each genotype, respectively. Boxes indicate the median (bold line), 25 and 75 percentiles (solid line), respectively. Dashed lines indicate the minimum and maximum of each quantified trait. (A) Allele substitution effects in NP-F_2_ progeny at 104-122 dph for genome-wide mapping. (B) Allele substitution effects in NP-F_2_ progeny at 149 dph for chromosome-wide mapping. The phenotypic values in N/N and N/P genotypes are not significantly different, while both are significantly different from that in P/P genotype (*P* < 0.0001, Kruskal-Wallis test and Dunn's test).

**
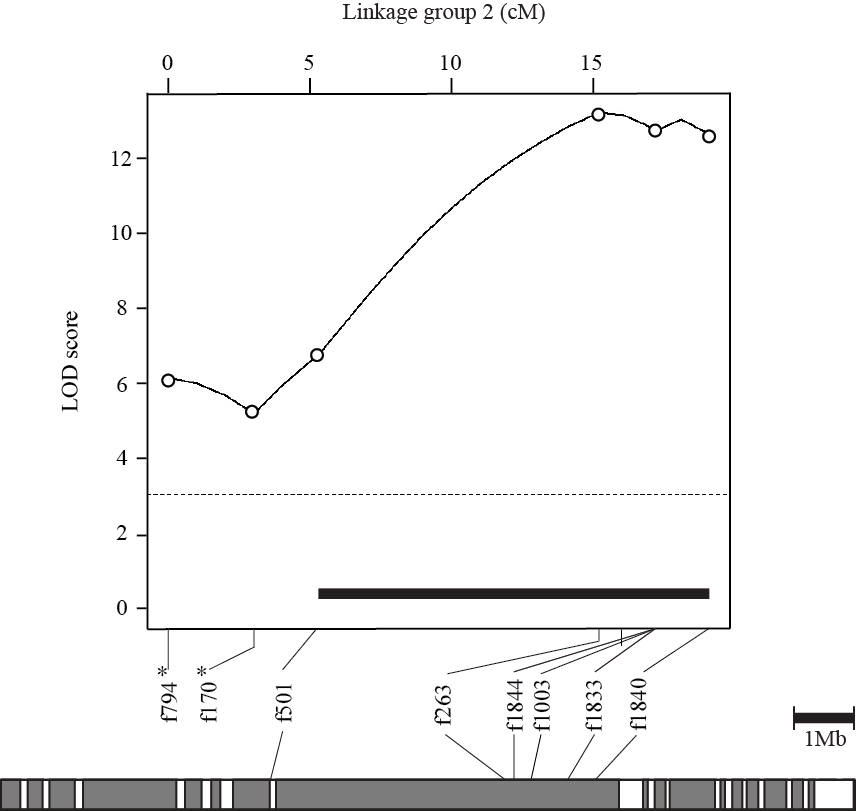
**

**Figure S8.** QTL mapping for scale reduction in NS-BC progeny at 42 dph using the binary phenotype data. Chromosome-wide QTL mapping plotted against linkage group 2. LOD score is on the y-axis, and map positions of markers on linkage group 2 are indicated under the x-axis. LOD curves and highly significant levels are shown by solid and dotted line, respectively. The chromosome-wide highly significant level calculated using a permutation analysis (n = 10,000) was LOD = 2.73 (*P* = 0.001). The black bar indicates the 95% credible intervals associated with QTL. Open circles show LOD scores of each marker. The gray bar under the LOD plot depicts the physical map of fugu chromosome 2. *Marker position on fugu chromosome 2 has not been determined.


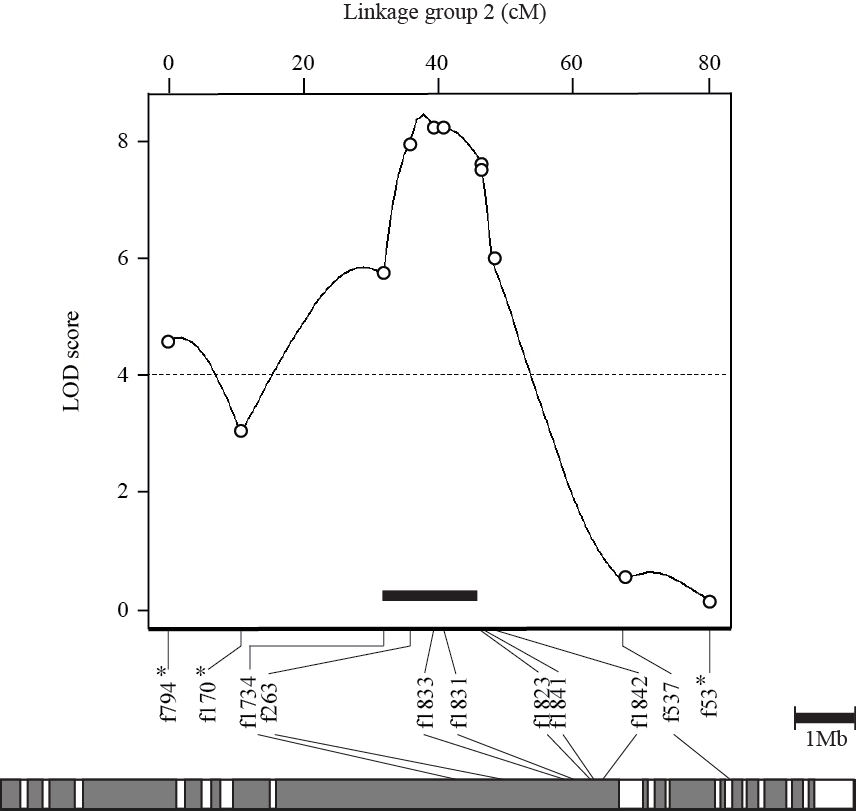


**Figure S9.** QTL mapping for scale reduction in NS-F_2_ progeny at 109-110 dph using binary phenotype data. Chromosome-wide QTL mapping plotted against linkage group 2. LOD score is on the y-axis, and map positions of markers on linkage group 2 are indicated under the x-axis. LOD curves and highly significant levels are shown by solid and dotted lines, respectively. The chromosome-wide highly significant level calculated using a permutation analysis (n = 10,000) was LOD = 4.01 (*P* = 0.001). The black bar indicates the 95% credible intervals associated with the QTL. Open circles show LOD scores for each marker. The gray bar under the LOD plot depicts the physical map of fugu chromosome 2. *Marker position on fugu chromosome 2 has not been determined.


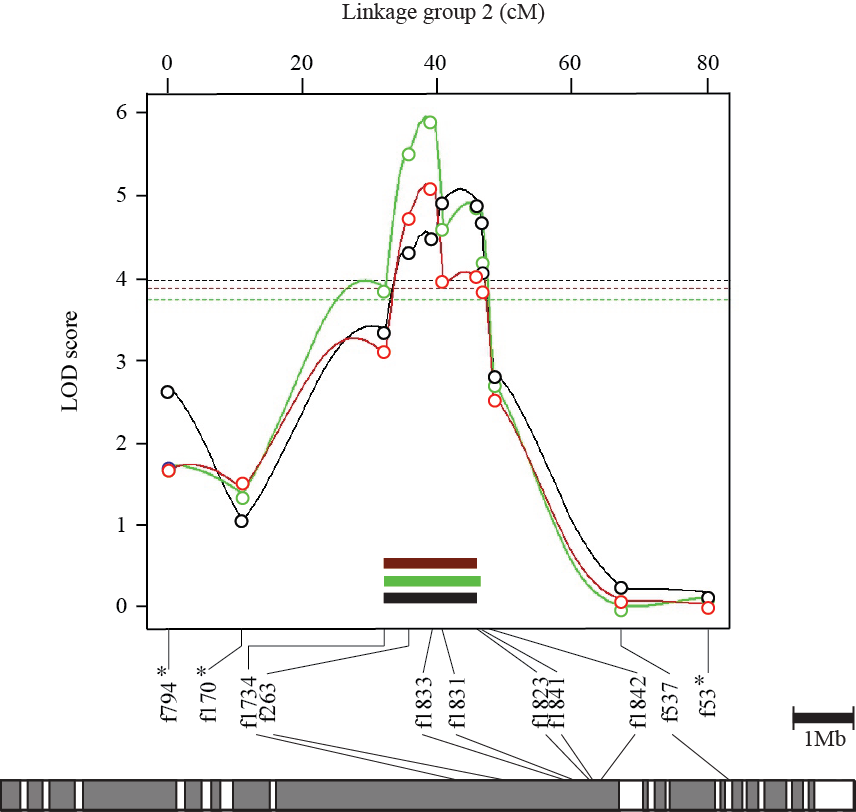


**Figure S10.** QTL mapping for the reduction of scales in NS-F_2_ progeny at 109-110 dph using the quantified phenotype. Chromosome-wide QTL mapping plotted against linkage group 2. LOD score is on the y-axis, and map positions of markers on linkage group 2 are indicated under the x-axis. LOD curves for the three traits (the total number, total area, and size of scale) are shown by black, blue, and red lines, respectively. The chromosome-wide highly significant level calculated using a permutation analysis (n = 10,000) was LOD = 3.95, 3.72 and 3.86, respectively (*P*= 0.001). The 95% credible intervals for the three traits are indicated by black, blue, and red bars, respectively, under the LOD curve. Open circles show LOD scores of each marker. The gray bar under the LOD plot depicts the physical map of fugu chromosome 2. *Marker position on fugu chromosome 2 has not been determined.

**
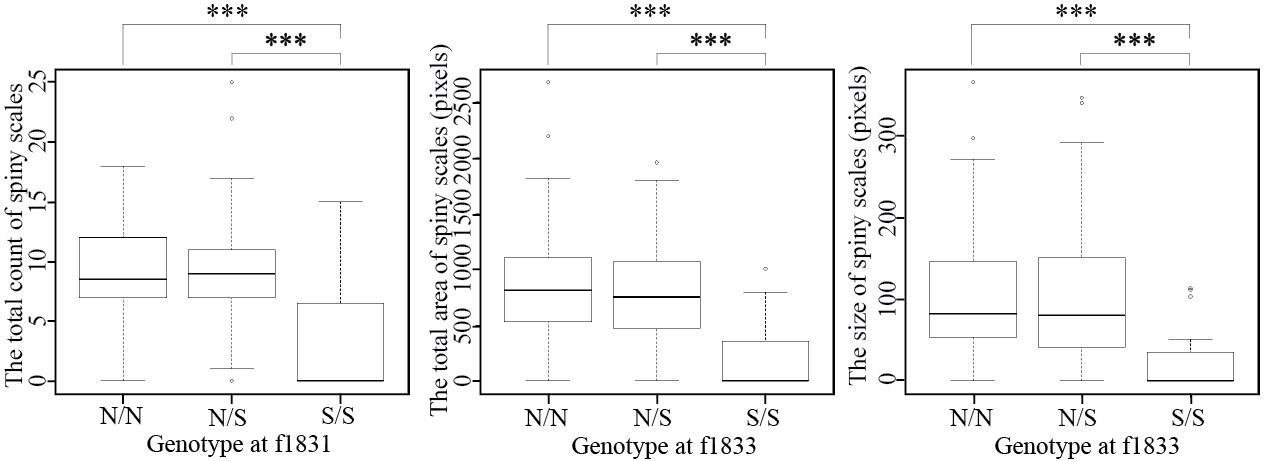
**

**Figure S11.** Effects of genotypes on scale variation in NS-F_2_ progeny at 109-110 dph for chromosome-wide mapping. The genotypes were obtained from maker locus nearest to the QTL peak. The allele derived from *T. niphobles* and *T. snyderi* are defined as N and S, respectively. Boxplot shows distributions of three quantified traits, the total count, total area of scales, and the average area per scale from each genotype, respectively. Boxes indicate the median (bold line), 25 and 75 percentiles (solid line), respectively. Dashed lines indicate the minimum and maximum of each quantified trait, and scatter plots refer to outlier. The phenotypic values in N/N and N/S genotypes are not significantly different, while both are significantly different from that in S/S (*P* < 0.0001, Kruskal-Wallis test and Dunn's test).
