# Supplementary material for: The Genetic Basis of Scale-Loss Phenotype in the Rapid Radiation of Takifugu Fishes
Source: Genes (Basel). 2019 Dec 10;10(12):1027. doi: 10.3390/genes10121027 (PMC6947334; doi:10.3390/genes10121027)
Supplement: Supplementary file 1 [file genes-10-01027-s001.zip › supplementary files revised191206/SuppleTable20191206.docx]

**Table S1.** List of the genomic position and primer sequences of each locus used for the linkage map construction of NS-BC family by means of target amplicon sequencing

| Marker | Foward primer (5'-3') | Reverse primer (5'-3') | Fugu chr | Amplicon start | Amplicon stop |
| --- | --- | --- | --- | --- | --- |
| 1313533_60 | TTGGAGTCATTTAAGGACACGGTT | GGACACATTTGTATGTAACACACGT | 1 | 1313510 | 1313679 |
| 4516789_31 | AGAAGAAGTAGTAAAGGTTTAAGAGGGTGT | ACAGGTTCTTTGGTATTGTTCAGCT | 1 | 4516760 | 4516934 |
| 5991741_31 | AAAGGAGGAAAAGAAGCATAGCAACTTA | TTAGCATGACACAGAACCATAGTGAC | 1 | 5991714 | 5991888 |
| 6334586_30 | AGATGTTGGGTTGGAGGGTTTTT | CAGTGTTTAAAGGTAGAATAGCTGCTACT | 1 | 6334564 | 6334738 |
| 7666039_31 | CGCATTTCCAACATGTCTCTCGA | GCTCCATGGATGAATCATCCTCA | 1 | 7666017 | 7666191 |
| 8215478_100 | AAAAAGTGTTGCGTTTGACATGTTTT | TCACCACTGACAGCTCTACCTATAC | 1 | 8215453 | 8215627 |
| 8546301_117 | GCTGTTGGGACGAGATTTTGCT | GTATAGATCCACAGTGACTTCAGTAATGG | 1 | 8546280 | 8546454 |
| 8960565_48 | TTGCTTGTATGCTAATTTGATACTCGACT | GTGGGCGTGTCTGAGAAAATTG | 1 | 8960537 | 8960702 |
| 10136070_72 | TGAGATGTTCCATGTTGCCACA | CCAGCACGGTAAAGTGTTTTACATTC | 1 | 10136049 | 10136223 |
| 10873560_122 | CGCTGGCTCAGCTGGAAA | GCTGGACAGACTGGGACAGTA | 1 | 10873543 | 10873710 |
| 11768874_70 | CTATGTGATTAGGTGTCAAATGTTCTTGTC | TCTAATGACTTTAAGGCCTTGAAGCA | 1 | 11768845 | 11769019 |
| 11969347_72 | ACTCATGTCGTCTGACCCACTA | TGAAATGTTTGTGCTTTTCTATGTGCA | 1 | 11969326 | 11969500 |
| 14289194_105 | CCACGCTGTGAAGACAGGATTA | TGTTTATACACCCAACAATTAGCTGCT | 1 | 14289173 | 14289347 |
| 14590847_57 | ATTAGAAGCCTTTGTTGCTTTGAACC | TATTCTGGAGGTGGTCTCTGGTT | 1 | 14590822 | 14590996 |
| 15556430_36 | CCGAGAGGCTTACCTCTACAAAC | ATTTTTCTTCAAGGTCTGCTTAATGACG | 1 | 15556408 | 15556582 |
| 17050366_60 | AAGGTGTGAGGAGGTCCACTAA | CTAGACTGTGTTTGCTCATTCAGGA | 1 | 17050345 | 17050519 |
| 17872269_44 | GACATTAAGGATAGCATCCTGCTGAT | GAGAACAAACCCACACAGTTCTG | 1 | 17872244 | 17872413 |
| 18135818_106 | GGCTCGACTACACTGACAATTGT | TGGATGCAGTTTTCATTGTTTTAAGACATT | 1 | 18135796 | 18135970 |
| 18790114_52 | CCAGAGGGTGAAGAACATGCTG | GGGATACTCGAAGGTGAAGTCGTA | 1 | 18790093 | 18790267 |
| 19753662_38 | GGAATCCTGTAATTACCATCAAATTGTGG | CCCAAAATAGAACTCCAGGAAGGAA | 1 | 19753744 | 19753918 |
| 20159307_50 | CTCGCCACGCTTGAGGAA | GGATCAAGAAAAAGAGACAAAACTACAGC | 1 | 20159290 | 20159437 |
| 20177779_73 | GCAGGCTTTAATGGATGGCTTT | AGCCATAAAGATGTTTGCACCTACA | 1 | 20177758 | 20177932 |
| 20503064_95 | ACTGCAAAGACCTTATTTTTGTTACATGAC | GCAATGCAAGACATGTTTTTAATGCAT | 1 | 20503035 | 20503209 |
| 21570363_44 | ACTGTATGCACTCTGTCCTCTGT | ATTCAGGGTGATATTTGCAATTCAGC | 1 | 21579341 | 21579515 |
| 22734039_84 | ACCTTTGGCTTCCATAACGTCA | CGTTGCTAGGCTATAAAACAGGCAA | 1 | 22734018 | 22734192 |
| 22782272_104 | CATTCCTTTCACGACACCAAGATG | CCAGTAGCTTAGTACCACCTGAACA | 1 | 22782249 | 22782423 |
| 708609_71 | CCACAAGTACCCAACCACGTTA | GCTCTCCCAGAAATGAGTCAGACT | 2 | 708588 | 708762 |
| 1250843_69 | AAGAGACACTTTAGCAGCTGAAACA | AAGGGTTTTCTTTTTGTACTCAAAGTTGG | 2 | 1250819 | 1250993 |
| 1897244_119 | TTGATGAGCCAAAGGTGGATGT | GCATTGGTCATTGTCTCTGCTTCT | 2 | 1897223 | 1897397 |
| 3241529_47 | GGCACACCTCGTCCAGTATATC | GGGAGGACTTCATGAGCAGAATG | 2 | 3241508 | 3241658 |
| 3433936_55 | CTGCAGCAGACGCGTCAGAAAT | CCCTCCCAAAGTTGGTTTCCTTTAG | 2 | 3433915 | 3434089 |
| 4084976_37 | CTTGACAGCTGGAGGACCTATT | CATGGGTGTAAGTCATGAGGTGTATG | 2 | 4084955 | 4085127 |
| 5032003_66 | AAACACACCATTACTCCATCAGCT | CTCTAACACCCATCTATCATCTGGAATG | 2 | 5031980 | 5032154 |
| 5236169_70 | AGGTCTGAGAAGTCAACAGGTGA | CAGAACCAACACAAAGGTTTACAGTAG | 2 | 5236147 | 5236321 |
| 6488104_75 | GTGCCCATTCTAACCTGTGTGT | GAGAGAATGACAGAATCTAAGAATGGAGTC | 2 | 6488083 | 6488257 |
| 7222201_17 | CCTTTTGGAATGGTTCGCAGAGTA | ACCAATGCGCGTTTAGTTAAATGATTAC | 2 | 7222178 | 7222352 |
| 10421496_81 | GAGTCATCCAGAACATGAGCAGAA | CGACTCCTTCAGCTGATCGATC | 2 | 10421473 | 10421647 |
| 1003319_64 | TATGCTGCTTGGTCTTGGAAATGA | AGAGTCTGAAAACCGAGGTTTTAACAG | 3 | 1003296 | 1003470 |
| 1135614_40 | TTCATCAGCAATCACCTTGAATTTAAGC | GATGGTGATGTGGTTGCTAAACAC | 3 | 1135587 | 1135761 |
| 1555911_111 | TCAAAGGATGATCTTCAAAATATGGCGT | ACCATGAGCTGCTTGTAAGTGAG | 3 | 1555884 | 1556058 |
| 2803401_35 | AGGCCTGAGACATATAAGTGGTCT | GGCACCTGGAGTGGTACAAATATG | 3 | 2803378 | 2803544 |
| 4142176_92 | GATGAAAACTGCTCCTGACAACAC | ACCAGCTTCACTTCCTGTTAGC | 3 | 4142153 | 4142327 |
| 5922940_53 | GCTGAACCATGTGATCTTTTGTCC | CGATTCAGCAAAATGTGCCAACA | 3 | 5922917 | 5923091 |
| 6407553_67 | TGAATATAAATAGCCCTGGCTGCTAAG | CTACTAGCCAGGAACACGAGCCT | 3 | 6407527 | 6407701 |
| 9446111_67 | CACCTGGTGGCCAGATATGATAT | ACCAGCAACTCCATGTACTACAAC | 3 | 9446089 | 9446263 |
| 9627764_27 | GATCTGCAGCTGAGAGAAGAAGA | CACTCTCAGTTATCGCCATAATAGTGG | 3 | 9627742 | 9627916 |
| 10273822_60 | CCGCTGGAGGTTCTGACA | GCACTTCTTCTACCTCCTGCATTTAAAA | 3 | 10273805 | 10273952 |
| 10947956_53 | GCTAAGCTAAGATTATGGAAATCAATGCC | CTGAATTGTTTGGTCACATACTATTGGAG | 3 | 10947928 | 10948102 |
| 1074728_43 | TGCAGAGAAAAGTTGCCTCTCTT | AAACAGCAGCTAGGATTAAAAACAAGC | 4 | 1074706 | 1074880 |
| 3229790_92 | GCATCAGCAATGTTTTAGGGCT | GAGACACGCAGTTTGGACATTAAAAT | 4 | 3229769 | 3229943 |
| 3764824_14 | GTATTTGTGATGCTGGGTTTAAGACC | GCTTCCGATTGTCCGTTTGTCT | 4 | 3764799 | 3764973 |
| 7418628_62 | CCGACCTCAGTGTCTCTCTGTT | CGCAGCCCTTCTCAGTTCTTAAA | 4 | 7418607 | 7418773 |
| 9623143_86 | GTACAAGGAACTGGGTCGAGTT | GCGAGGCTTTGTCCTTCAAATC | 4 | 9623122 | 9623296 |
| 10108609_62 | GCTGGTTCCAGAACAGCCTTAA | GCTACACAGTGTTCCAGCATCT | 4 | 10108588 | 10108740 |
| 11688463_102 | GTATCAGCTGGCTGTTGTTGGTATA | TTGGCTTTGAGGTTATTTTAATCTGGTAGT | 4 | 11688439 | 11688611 |
| 12096785_14 | GATCATCCTATGACAATGGCATGACT | TAAAATGAAGGGAAGCTGCCATCA | 4 | 12096760 | 12096934 |
| 1258491_39 | TGAACAGGAACCAACAGAACATCTT | TGAACAGGAACCAACAGAACATCTT | 5 | 1258467 | 1258641 |
| 2583591_107 | CCAGTGTTCCTTATGGTCGTGA | CTGGTTATGATCAGTGAGTGACCAAAA | 5 | 2583570 | 2583744 |
| 3521802_115 | CTCCATTTCTGCTCCTTCACATTTTC | CCATTTCTAGCAGAGGTTACGCTT | 5 | 3521777 | 3521951 |
| 7818719_25 | ATTTGCATGATGCATTGATCGCA | GTGTCATCGGTGCAACAAACTG | 5 | 7818697 | 7818863 |
| 8362451_77 | AGTTCCTCCTGTAACGTCAGTGA | CTTTCCCATCAACTTTGCAGAATGTTTAAT | 5 | 8362429 | 8362603 |
| 8909218_37 | TGTGAGTCGTGTGCGTGAAA | GGGACACTTGATGAGTTATAATTAGAAACT | 5 | 8909199 | 8909346 |
| 133940_35 | TGCTAAACAAACAATATCCAAGGACATCA | ACCAGAAGATGACAGCTGCAAAT | 6 | 133912 | 134086 |
| 396728_31 | CATTTGTGTGTGCTGGAACTGT | CCTGGCATTGAAATGAATTACAAAGGG | 6 | 396707 | 396881 |
| 1057493_24 | GGACTTTGAGGGCCACTAGAGT | TGGAACGTCATTTTGACATTTCTCCA | 6 | 1057472 | 1057646 |
| 1840714_91 | AACATCATGGAATATATGGGAGAAACATCC | GGAGTTGTAGCCTGAAGAAGAAGAATAAAT | 6 | 1840685 | 1840859 |
| 3840378_50 | GGAAAAATTAGAAGCGATTGAATGGCA | TGGAAGACTTTGATCTCAGGCACTA | 6 | 3840352 | 3840526 |
| 4330020_82 | CTTCACCTTCTCAAACAGAGCCTT | GAACGGCTCAATGGACAAGAAG | 6 | 4329997 | 4330171 |

| Marker | Foward primer (5'-3') | Reverse primer (5'-3') | Fugu chr | Amplicon start | Amplicon stop |
| --- | --- | --- | --- | --- | --- |
| 6647897_12 | TTCACAATGCATTATGGGAAACAATGAG | ATGGACACAGAGCAGTAGAGACAG | 6 | 6647870 | 6648044 |
| 7890657_89 | TGTGCTACACTCATTTGCACTGA | CTCATCTCCATCATATTCATCCCGTAAC | 6 | 7890635 | 7890809 |
| 8489770_39 | TGCTGAACTTGCCCACCA | GAGGACAGAGGTGAATCTCACTG | 6 | 8489753 | 8489927 |
| 8641672_106 | CATTTTACAGCTGTTGATGATCAGCA | GCGAGCTTTTAAACACAAATGCATCAT | 6 | 8641647 | 8641821 |
| 29371_59 | AGCAACAAGGATACAAAAACATGTCTG | TGACCAGTCTATGTGCTTGGTTTTT | 7 | 29345 | 29519 |
| 1381813_68 | GCCAATAATGGAGGGCAGTAATAAATCC | ATAGCTTTCCCACCTTAAGACCTTTAAC | 7 | 1381786 | 1381960 |
| 4309247_76 | GGACGTGCTGGAAGAGGAAG | GTGAAGCTCCGTGTTGGTGA | 7 | 4309228 | 4309402 |
| 7131439_103 | ACCAACTAAAACTGAGGAAAGTCAAGT | CCAACCCTCAGGATGGTAAATGTTTTATAT | 7 | 7131413 | 7131587 |
| 7249646_73 | GCTTCGGCTACAAGGAGTCAAA | GTGTCGCTAACATAACAATTGCTTTGT | 7 | 7249625 | 7249799 |
| 8039700_47 | GTGCAACTTCTTCCTGGTACCAA | CCTGAGATTGACAGCCTTGATGAG | 7 | 8039678 | 8039852 |
| 8096666_114 | TGAAACTAATTACCGGTGGATTTGCT | TTCAGGTGGGAGAAACAGACACAAT | 7 | 8096641 | 8096815 |
| 9199304_95 | TACATACCTCACCTCCCACTAATAACAA | TGCTATTCAGGATAATCAACATGTGTTTCT | 7 | 9199277 | 9199451 |
| 9325973_70 | CTTTTAAAACCATGTTCCAGAGATGGTTG | CCTCTACTTCCTGGTTTGGCTT | 7 | 9325945 | 9326119 |
| 10297429_36 | CTCCCAGGCTCCTGTATGTTG | CCCTGAGGAAACATTAGAATTCTTGAAG | 7 | 10297409 | 10297577 |
| 11634549_50 | CATTGACAGTCCACTGGGAAGA | AACACTCAAGGGTAATTTATCTCGGTTTAT | 7 | 11634528 | 11634702 |
| 12108185_31 | CTGTTTGAACCTGAATGCTGGT | CCTCTCAAGCAGCAGCTTCA | 7 | 12108164 | 12108336 |
| 13111649_37 | GCCTTTTGTGATGTGTGCTCAA | ACTGGGACTCTCTGGTTTGTTAAC | 7 | 13111628 | 13111802 |
| 459614_108 | TACTGTTTGTGAAAGTGTCTTCCATGT | GATAACTGAACCAATCATTCATCACCAAG | 8 | 459588 | 459762 |
| 2628420_65 | AGGCCTAAAAGTCTCTTAATTAGACTCCA | TCAGAGGATATTTTTGAGGATAATGACACC | 8 | 2628392 | 2628565 |
| 4324825_43 | CTTTAGTAAAGAGCAGCATAGGCTGA | GATTCAAACATGAGCATTTAAGGAACACA | 8 | 4324800 | 4324974 |
| 4821474_12 | CTCAGGATTCTGCGGTAAAGCA | CACAAAGTCAGACTGGTTTGATTCATG | 8 | 4821453 | 4821627 |
| 4953724_98 | GCCTTCAGCAGCAGATTATGTG | CCACCTCTAACTTCTATCCCTTTGTG | 8 | 4953703 | 4953877 |
| 5128125_96 | ATTTACCTAGTTGCTGCTGGCA | CAAAAACAATTCAGCAGTATTCAGATTCTACAG | 8 | 5128104 | 5128275 |
| 5228698_118 | CGTGTTTTTGGCAGGAAGACAT | ACATAGTCTGCAGCTGTTTGGT | 8 | 5228677 | 5228851 |
| 5675340_101 | CCCGTCATCCCACTTAAACTATGA | CACAGATTAGGTGGAAACACATTGG | 8 | 5675317 | 5675491 |
| 6957427_113 | CACAAGGTCACCGAGAAACCTT | CCCTGTCCTGACTTGTCCC | 8 | 6957406 | 6957572 |
| 7377004_96 | AGCAGTTTATGCTAAAACAGCAGTT | CACCTGAGCCAGATTAAAGATCACA | 8 | 7376980 | 7377154 |
| 8563133_106 | CCATCCACAAGGTGAACAGCAT | GCTCATCAAACGGAGGGTAGAAA | 8 | 8563112 | 8563285 |
| 1064104_106 | TTGGATTTCTACCAACAGGGTTTAATCA | TACACCAAGGCACTAGCGAGTA | 9 | 1064077 | 1064251 |
| 1724342_100 | GCACATATGAGTACTATGAACCGTAGT | GCTGTACATTAAATATGGGATGAGAGCTAA | 9 | 1724316 | 1724490 |
| 1957021_51 | TCCATCCATCCCGAGACAACATA | GCGCAAAAGGACCCGTGTAATA | 9 | 1956999 | 1957167 |
| 3184710_82 | GGACTCCAAGTGAGAGACAGACA | GTGTCCAGGGAAGAGACAACCA | 9 | 3184688 | 3184862 |
| 3318270_14 | ATTTTATCATAACCGTCATCCAGTGGAA | CTCTCAGCTCTCCACCTCTCAT | 9 | 3318243 | 3318415 |
| 7478254_79 | TGCTAGAGTTGCTCCAGAGTTAAATC | TTGGTGGAAACATTCAACATGTATCTCT | 9 | 7478229 | 7478403 |
| 7918633_101 | CTGCTGGGCTGTTCAATCAAAG | TCTGGTTTGTTACTGATGTCGCATT | 9 | 7918612 | 7918786 |
| 9915399_52 | GTGATGTCGTTAGGACATTAAATGGC | GGGTACTTCCACACAAAGGTGTAAAA | 9 | 9915374 | 9915533 |
| 10607335_82 | GCCGAGCAAGTGTTATTTATTCACTC | TTATGAGCTGAGAGGAACGACATTG | 9 | 10607310 | 10607484 |
| 11782311_108 | TCCCATCGGAGATGCAAAACAG | TTTAATACTGCGCGTCAAGTTGTTATTTT | 9 | 11782290 | 11782464 |
| 11887840_114 | GATTTTGTTATTCCCAGCCGTCATT | TTGATGTACAGCCTGCTCAACATAA | 9 | 11887816 | 11887990 |
| 11912287_13 | AGGAGCGGCTCATTAAGAGTAAAG | TCATTAGCGAGGTCTTTTTGTATAACTGG | 9 | 11912264 | 11912438 |
| 619593_35 | CGCAGAAGAAGAATAGCTGGCAA | TGTGCCATGAGGAGCTATGAAATT | 10 | 619571 | 619745 |
| 817085_29 | CCTGAAAGGGTCCAGTTGGATT | CTGTAAAAACACAAATGCAGGTTCGAT | 10 | 817064 | 817237 |
| 3160805_12 | AGCATGTTGAAGCTTTTCTTTTTAGTGT | ATCCTTGACAGTGTTCTTATTCAGTCAG | 10 | 3160778 | 3160952 |
| 4038462_68 | TTGTGTATGCACATGTCTTGTTTGTG | GGCCGTAAACAGCTCATTTAGC | 10 | 4038437 | 4038595 |
| 5795293_84 | GTAGAATCATAAAGGAGAACAAGCAGAGA | TCACTTCTATTGCTAAAAAGGTCGGATT | 10 | 5795265 | 5795439 |
| 6672536_42 | CTTCTTATCAGCCTTTCCCGTTATTTATCT | ACTCTTTCCTGAAAGTTATTCCGCA | 10 | 6672507 | 6672679 |
| 6863714_105 | CAATCTAAACTTTGAGTGATGGCACTT | CACTGGTCGTGTAATGTAGGTTAGTG | 10 | 6863688 | 6863862 |
| 489410_18 | CCGACGCTGATTTCTCACATCA | TGTGTGACATTGTTTGAAGATTCAACAAC | 11 | 489389 | 489547 |
| 1671870_73 | CCCTGCCCTGACAGTTAAACTA | AAATTGACCTGTAAAAGCCATTTAAAGCAT | 11 | 1671849 | 1672023 |
| 3833715_100 | CTCTGATGCTGATGGGTGCATA | TCTCTCCTGGATTTCAATTTTGACACA | 11 | 3833694 | 3833868 |
| 7001073_54 | GGATGCGGGTCCATTAGAACTC | AATCATCCATTTATGAGGTGACTACTCAAC | 11 | 7001052 | 7001204 |
| 7227419_57 | GTCCGAAAGGAGGATAATTGCAAC | ATTCAAGCGATCCGTCTTATCATCTC | 11 | 7227396 | 7227570 |
| 7902927_102 | GAAAATGGAGCAACAATCATCCAGT | TGTGGCAATTTTAGCAATAATTAACTGGTC | 11 | 7902903 | 7903077 |
| 9870540_56 | GGGTCCGGATAGCAGAAACTAG | GGAAGCGCAAACTATTTAAGTGCATTTATA | 11 | 9870519 | 9870693 |
| 10568362_85 | CGAGCATCACGGGAATCGA | GTGTGCGTGGTGCGTTTT | 11 | 10568344 | 10568510 |
| 122543_11 | GTGGATTATTTGTGTATTTTTAGCCTTGGT | CAGACGATGCTAGAAAAGACAGTGA | 12 | 122514 | 122688 |
| 3269813_66 | CCAGGTTTAGGCCATGTCTAGC | CCTGCCTTTTCTTTATAAAACGGGC | 12 | 3269792 | 3269966 |
| 3430192_23 | CCCGCTTCAGACAAAAGCAATA | CCATGGGTGTTTGTGGTCATCA | 12 | 3430171 | 3430331 |
| 3468744_25 | GTCCAACATTCTATTCAGGAGCTTCA | CTTCAAACGTCCTCATAGATACAGTAAAGT | 12 | 3468719 | 3468893 |
| 8069333_32 | AGTTTAGAGCTTATGAAATGTGCTGAGA | TTCAGCACAGCTACACTTGTCA | 12 | 8069306 | 8069480 |
| 8080821_35 | GTGCCTGTAACACACCTACAGA | GTGCCAAATTTATAGGAATGAAAAGTCTGA | 12 | 8080800 | 8080947 |
| 8235039_28 | CAGTGACATGAATAAAGGCGAAAACA | TACAGCTAAGCTTGTTTGCCTCA | 12 | 8235014 | 8235188 |
| 8304875_62 | GGGAACTAACATCGCTACAGCAA | GGCCACTTTGAAAGAGGAGGAAAT | 12 | 8304853 | 8305027 |
| 8342793_112 | TGCGTCTGAATTAGACCAAGCA | CAAATCGTGAACCAGCCTTAGAATC | 12 | 8342772 | 8342941 |
| 8837752_99 | TGCTCGGCTACACCATGTG | GTGACGAGCAGCTGCAAAC | 12 | 8837734 | 8837908 |
| 1429041_65 | TGGGTGTGAAATGTGTCAAAGTGT | GCGTTGTCTAATATCCAAATTAGCAAAGTG | 13_1 | 1429018 | 1429192 |
| 2742136_19 | CCTTTCCTTCTCTATCGCTTCTCA | CAGTGACCTTAATGTGTGCAAACAC | 13_1 | 2742113 | 2742287 |
| 2896445_109 | TAACCATCTGTAACCAGCAGATTTCAG | GTTGTTCCTGTGGTCATAAATGAACCTA | 13_1 | 2896419 | 2896593 |
| 4642361_25 | CAGAGAAATTATGGACAAGGTTTATTCCCA | AGAGCAGTTTAAAGGACCTAAACATCC | 13_1 | 4642332 | 4642506 |
| 5294529_30 | CCCTTCACATGGAAAATGAAAACTGT | TGTCGCCAAGTAGTTTCCCAAG | 13_1 | 5294504 | 5294660 |
| 6666190_19 | AAAACAAACTAATCATCCAGGCGTAAATG | GTCTCACCTTGCAGTAGGTGTTT | 13_1 | 6666162 | 6666336 |
| 8508059_33 | CCTGAGCATCAGCCTGTAGAAG | TCCGACCGCCAAATTCATTTAAATC | 13_1 | 8508038 | 8508212 |
| 8992336_128 | GTGCATTTGGGATGAAATTTGACG | CATGCCCAACTAGCCTCTGA | 13_1 | 8992313 | 8992487 |

| Marker | Foward primer (5'-3') | Reverse primer (5'-3') | Fugu chr | Amplicon start | Amplicon stop |
| --- | --- | --- | --- | --- | --- |
| 9859442_54 | CGTGACCTTCCCTGTCTAGACT | GCCATGTGTAGAGATTTATAGGAGATGAAA | 13_1 | 9859421 | 9859595 |
| 13511192_115 | GCGCCTAGCATCTCAAAGACAA | CCTGCAAGCTGAGGACTTTTTACT | 13_1 | 13511171 | 13511345 |
| 16479637_61 | CCCTCACATCAGAGAGAGACACT | CGCCGTTTATGATCACGCAATAG | 13_2 | 16479615 | 16479788 |
| 16670275_81 | CGGTAGCTCCTGCTGGTGTATA | CTAAAGATCTGTACAGAAGAAAGAAGCAGT | 13_2 | 16670254 | 16670415 |
| 332903_69 | CAGGCAGCTCCAACTCATTACT | CACCATCACTTAATAAAATCAAAAGCAGCA | 14 | 332882 | 333050 |
| 1445757_110 | ACCGTGTGAATTTGAGCCTGAT | CACGCATCTCTCTCATGTGTTTTC | 14 | 1445736 | 1445910 |
| 3973389_55 | TGTCGGTGAGGTCATCAGAACT | TGATGATGTTTAAAGCTAAAGATGGTAGCA | 14 | 3973368 | 3973515 |
| 4046532_25 | TCTCCGAAGTCCTGAGGGTTTTA | CTGCTTATTACAATGTCTCTGCCAATTAC | 14 | 4046510 | 4046684 |
| 7714536_41 | TGGACTTTGGATTCATTTTCTGTGGT | ATGTGAGACAACTCAGTCAAGACAG | 14 | 7714511 | 7714685 |
| 8156097_49 | ACAAGGTCACATGGGCATTATCA | TATACCTGACACTGACATTGGTAGCT | 14 | 8156075 | 8156249 |
| 8673679_107 | TGGCCATTCACACGTAACGTAA | ACTTTATAAAAGCTCAGCTCCCAAAACA | 14 | 8673658 | 8673819 |
| 9233921_82 | CAGCTACACCTTTAAACTGACCTCT | ACGCTGTGGTGTTTATTTTGGG | 14 | 9233897 | 9234071 |
| 11497437_15 | GAAAACAGCAATGCTATTGTCTGCA | TGGGTAGAGACAATGGGACAGTT | 14 | 11497413 | 11497580 |
| 12174325_100 | ACCAGACTCTGACTTTAATAAACACTCG | CGTACGAGCGGAAAAATCCCAA | 14 | 12174298 | 12174470 |
| 1282791_83 | TAAGAGCTGAAGTCAAACAGTTTCGT | CACTGTTCAGGGTAGCATGAGT | 15 | 1282766 | 1282940 |
| 4091770_16 | CAGCGCTAACGTGCTAACAAAG | CTGATGTGACCTTTGCCACGATA | 15 | 4091749 | 4091913 |
| 7177425_126 | GGAACAGGAGCCCTGTGTTT | GTCTCAGTGCAAGTGAGGTGA | 15 | 7177406 | 7177579 |
| 10414363_24 | CCACGTTGGCATTCTCCTCTAC | GTGGTGGAGAGAAATACCTTGACTTT | 15 | 10414342 | 10414516 |
| 208719_17 | TGAGGAAGCTTTCATTCATCCCAA | CATTTGGAATGTGGTGGCACTT | 16 | 208696 | 208870 |
| 1278141_89 | CCACAGCCAAAGATGGACTGAA | CATTTTTGGGTGTGGTGGTGTT | 16 | 1278120 | 1278294 |
| 1614533_72 | TTGTTTTTACTCTTGATGTTAGGGAAGCT | ACACAAATCACTGACAGCAACAAC | 16 | 1614505 | 1614677 |
| 2443506_45 | CATTAGCCTGAGCAGCGTTTAC | CATGATTCAGTTCTGTTGAACTCAACAC | 16 | 2443485 | 2443659 |
| 2741541_74 | GCTTTAATAGACTGTGGTGGAGGTT | GCAGCACTCACAAATGTGTCTTTT | 16 | 2741517 | 2741691 |
| 3068024_24 | ATGCAACAATGCTCATGTGCAT | TGTAGGTGCAAATGAACAACAAACC | 16 | 3068003 | 3068173 |
| 4292250_102 | TTGAGGGAAGGTTCCCAGACAT | CGTGTGTTGCTGTGGCATTTAC | 16 | 4292229 | 4292398 |
| 5075734_39 | ATGAGGACTGGGCCATGAAAAA | CCATCAGAGAGCCATCCTTCCTTA | 16 | 5075713 | 5075886 |
| 5311354_101 | GGATTTCTCACAGTTGGCTCCTT | CTGTTAATTTGAAGCAATGGGTGCTAAA | 16 | 5311332 | 5311504 |
| 5365072_64 | GCTTCACCTCCTGCTCTTCAAA | CACACACACACACACAGTCTTTAG | 16 | 5365051 | 5365194 |
| 6172837_78 | TGGAACATCAGGACTTTGGAATACTTC | TACCAAATCCAGCATCGACAAGTAC | 16 | 6172811 | 6172985 |
| 7148736_29 | TCCGCTTCATCTAGGTGGATCT | GTTCATTCAAAGGTACAGTTGTTCATGTT | 16 | 7148715 | 7148889 |
| 10207654_109 | AAGAACCGAGACAGTGAATGTTTTGATA | GGGAAGAGGATCCCATAAAGAGCT | 16 | 10207627 | 10207801 |
| 2215442_122 | AGTCTTCCTCAACCTCCAGTCA | CTGGACTTGTTCAGCCCATGT | 17 | 2215421 | 2215595 |
| 2491165_61 | CACAAAGCTGGCTACCAGTGA | TGACCTGTTATAAACAATAAGAGATGTAGC | 17 | 2491145 | 2491316 |
| 2911262_40 | CATGAGCTGGTATCGGATCTAACAA | AAACATTCTGCCATATAATCACCCTGATT | 17 | 2911238 | 2911412 |
| 7120942_19 | CGGACTGTTAGTGATGCACAAC | GTTATATTTGTGGTTGTTTGGAGCCAT | 17 | 7120921 | 7121095 |
| 8888232_82 | AACCTCAATATCCTACGCGTTGATTT | TCATGACATTTGCTGTATCGCACT | 17 | 8888207 | 8888381 |
| 1332459_57 | GTTCTGCACGAGTTGTAGACGA | TCAGCTCTATGTTCAAAATGAGCTTCTAC | 18 | 1332438 | 1332612 |
| 1998117_117 | GCGGGAGATTTGAACGGTTTTC | GCTGTCAGCAGAGGTTTGAGAG | 18 | 1998096 | 1998269 |
| 3837118_26 | GGACAGCTGGGAGTGAGATTTG | GGCAGGACTTGGACAGAGAGTATTAT | 18 | 3837097 | 3837257 |
| 4203169_110 | TAACTGCGCACCAAGTAGGAAAA | GGCAGTTTGAAGCAAATTTCGC | 18 | 4203147 | 4203321 |
| 4753812_89 | CAGCATGCCTTTCTCTGTGTTT | GCATTCAGATGTTGCAGTAAATTCCAC | 18 | 4753791 | 4753965 |
| 5521705_49 | CTACTCCCTCTTACTTACACAGAGGA | TATCACTGTTACTGGCTGTTAACAGC | 18 | 5521680 | 5521854 |
| 6086916_44 | AAACACGGGTGTTCGAGACAATA | GGCCACGTGCAAAAATCAAGAG | 18 | 6086894 | 6087053 |
| 6941421_15 | CACTGACAAAACAACACAAGAAAACTACT | TTCATGCCTACTAAGTGGAACATGAC | 18 | 6941392 | 6941566 |
| 7875196_34 | TGAGGTGACAGGTACAGGACAA | CCTCTTGTCCTTGGACTCACATG | 18 | 7875165 | 7875304 |
| 860790_26 | ATAATCACGTAATGTGCGGAGTGAA | TTAGGTTTGGTTAGGCGTTCTTGT | 19 | 860766 | 860933 |
| 1301242_27 | GGATTAGATGGAGATTAAATGTGAAGAGGT | ACAAACTTGCTAGTAGACTGAACAGTTT | 19 | 1301213 | 1301387 |
| 1645693_57 | GCAGGTGCTCAGTGAAAAATAAGG | GTGCAGCTACAGTCAAAGTGAAAATAAG | 19 | 1645670 | 1645844 |
| 2064671_44 | GGTGCTTCACCTGTGATTCTTG | AGACAAACAGACTCCTGGATGAAC | 19 | 2064650 | 2064824 |
| 2096481_86 | GTGTTCCTCCCTGACACACATA | TACCAATGCTGGATGGAAATAAATCTGT | 19 | 2096460 | 2096634 |
| 2230963_111 | CGGATTATGTCAGCACAGGGAAA | TGTGAGTGTGTGTTTGTGCATG | 19 | 2230941 | 2231115 |
| 2323001_123 | TCTGAACCATGTGAGGAGTTAGGA | ATCCAGCCGGAAACAGCACTAAAGAA | 19 | 2322978 | 2323152 |
| 2987775_70 | CAGTTTTGCTTCCTGTGATGGATG | GCATACAGTAAAACAAATCTCAACTTAGCA | 19 | 2987752 | 2987924 |
| 4775621_65 | GAGAAGGTGTCTGACACAGCTA | TACGCGACGATGCTGTGATTAA | 19 | 4775600 | 4775767 |
| 6555705_34 | AGACTGTTGGAGCACAGGAAAC | TCGCACTGATGTCACAAAATGC | 19 | 6555684 | 6555858 |
| 7147394_79 | CTGACTACTCCCTGATCTCTCATCT | CAAAGCCTGTGCACACTTGATG | 19 | 7147370 | 7147544 |
| 7451672_16 | AACACCACACTGTAATCTCTGCA | TCCATTAAATCCTGCCCAAATGTGA | 19 | 7451650 | 7451824 |
| 7705913_65 | AATCACGAAATAATGCAGAAAACTTCTGG | CTTGGAAGATCAGCTACAATGATACTCC | 19 | 7705885 | 7706059 |
| 9190916_45 | CATATTTGACAGGGCTGTAGTTGGA | GCTTCGGGAACTGACTTTTTCTATTGT | 19 | 9190892 | 9191066 |
| 11639183_71 | TTCAGCCGTTATCCGCCTAAAA | GACGAGGGAGAATTCATTTACCTACC | 19 | 11639162 | 11639336 |
| 12581458_20 | GCATGTCTTCAAGGATGCCAAA | AGATTAACCGCCTGTTGTTCCA | 19 | 12581437 | 12581611 |
| 254654_88 | TATGGTGTGATTGGTTATATAATGCAGCA | GCCCAACCCTAGCTTCAATGTAAATTTA | 20 | 254626 | 254796 |
| 2189297_31 | ACAAGAGACCGAAGCGATTCTC | CCCACTACCATCACATCACAAAGAC | 20 | 2189276 | 2189450 |
| 2784819_48 | GAGGGTCATCGAGTCTGTAAGC | ACTATCGCACACACACAATCTACAA | 20 | 2784798 | 2784972 |
| 4044886_92 | GCAGGCAGTGACGTGACTATAA | CGTCATCCAGTACCGCAATCAG | 20 | 4044765 | 4044937 |
| 4923471_76 | CTCCATCAGGCTCACGTTGTAT | GTGGCATCCAGATTCGGTAGTAAAA | 20 | 4923450 | 4923623 |
| 5149073_13 | GTCACGAGGAGGAAAACGAACAATA | CAGGACGCAGCTTTTTCTGTTT | 20 | 5149049 | 5149215 |
| 6338364_66 | AACAGCAATTAACATAATAACTGCAAAGGA | ACTTGCTCTCTGAGCTGTGTGATATA | 20 | 6338335 | 6338499 |
| 6799155_58 | CTTTACGTTGACACGCATGCTA | ATCCAACAGGCAAGGCAGGAAATAAAAC | 20 | 6799134 | 6799308 |
| 6940197_72 | TTCTCCTTGATCTCCTCTTCCACA | TGGTGTTTCAGGGAGAAGAGGT | 20 | 6940174 | 6940348 |
| 7334102_24 | CACAGATGGAGAGGAAACAGTCA | ACCACACACCTGATTAAAACAGGAA | 20 | 7334170 | 7334344 |
| 10050809_98 | TTGCTCAAGGTTTCTCTTGGCT | CTGGTTTGATATGGCGTTTTACAGG | 20 | 10050788 | 10050962 |
| 10962299_54 | ACCCACAAAAACACTTTTATTCACACC | TAGCACAACCTCTGCAAAAGCT | 20 | 10962273 | 10962447 |

| Marker | Foward primer (5'-3') | Reverse primer (5'-3') | Fugu chr | Amplicon start | Amplicon stop |
| --- | --- | --- | --- | --- | --- |
| 12300005_99 | AGAATGCTCGAATTAGCCCACAA | GTTTAATGTGCGCTTGTTTAGTTTTTCTTT | 20 | 12299983 | 12300157 |
| 13030583_92 | CGATGACCTTTCTGTGCACAATG | TAAGGCCTACATGGAAACTTTGTGG | 20 | 13030561 | 13030734 |
| 13324716_73 | TCTGGTTCTATTTCTCTGGAACATCTACT | CTGAGGGCTATAACGAGAAAAATAACAAAA | 20 | 13324688 | 13324862 |
| 1735367_11 | CCTGGAGACAGTTGCACATTGA | TTCTTGGTCTGGGCTACTTTCTG | 21_1 | 1735346 | 1735520 |
| 7405103_92 | GCGCTCTGTTTAATACTGTAGTTTCTTG | ATCATCTTGTCAGACTTTGGCAGATAG | 21_1 | 7405076 | 7405250 |
| 8399587_54 | GGATCAGGTGTGCTGTGTTTTC | TGTTGGAGTGTGTAGATTCAATCACATAAA | 21_1 | 8399566 | 8399740 |
| 12387134_52 | CTGCTGTTCCAGGTAAACGATTC | GCCGGAACGTCTTCATTTTAAATAATAGAT | 21_2 | 12387112 | 12387286 |
| 12870063_57 | CTTTTCATGTCAGCGTGTCTGT | GGCCTGGAGGCACAATATCATG | 21_2 | 12870042 | 12870216 |
| 13015487_81 | CTTCAGGACCTCTGGTGGAAAC | TTCAGACGGCACCTGAGAATATG | 21_2 | 13015466 | 13015640 |
| 14421470_29 | GATCCACGAAATTACTACAACAGGAAGA | GGAGGATATCCAGGAGCAGATGT | 21_2 | 14421443 | 14421617 |
| 14570844_65 | GAGAGAGAGATAAAGGAAAGCAGGAATG | GGGTGGCGTCTGTATGAGTG | 21_2 | 14570817 | 14570976 |
| 1976151_60 | CATGTATGTCAGCAGTATCTGTTGC | TATGATACTGGCACCACAAAAGACAAAT | 22 | 1976127 | 1976301 |
| 3374070_94 | ACTGCGGACGTTCTGCTTTATT | CAAGAATTTATTTCAGCAATGCCAAGACT | 22 | 3374049 | 3374210 |
| 3802884_57 | GTAGATGTGCAGAAGATGAAGGCT | AACTTTTGTGGTACAGATTTTTGTGGAAAT | 22 | 3802861 | 3803035 |
| 7917659_91 | AAACCTTTCGATTTCTCTGAATTTTACTGC | TGTGTCAGGTTTCCTGTGCTTT | 22 | 7917630 | 7917799 |
| 9587226_46 | GAACTTCTTGGTCACCTCCAACA | TCGTCGAACCTCTTTCTTAATGTTCG | 22 | 9587204 | 9587378 |
| 10487999_72 | ACAGGAAACGGTAATTCGATAAAGTGG | ACACAGTCACAAAGCTCCAGTATT | 22 | 10487973 | 10488147 |

**Table S2.** Information of microsatellite marker in NP-F_2_ progeny at 104-122 dph

| Marker | Foward primer (5'-3') | Reverse primer (5'-3') | Fugu chr | Polymorphisms^a^ | | | |
| --- | --- | --- | --- | --- | --- | --- | --- |
|  |  |  |  | ***T. niphobles*** | ***T. pardalis*** | **F_1_** | **F_1_** |
|  |  |  |  | **granddam** | **grandsire** | **dam** | **sire** |
| f619 | TGCTCCCAGATGTAAAAATG | CTTCAAGCCCACCAGTATG | 1 | Het | Het | Het | Het |
| f282 | GAATGCAAATATTGGTGCTCAA | ACAGGAAATGGCCTCAAAAA | 1 | Het | Hom | Het | Het |
| f330 | CCGCGACACCTCACCCATCAC | GTCCCACTCCCCTTCATCTCTTCA | 1 | Het | Het | Het | Het |
| f1712 | ATCTCTGAAGCCCAGGGAAG | CAAACCTGCTCAGTCCAACA | 1 | Het | Het | Het | Het |
| f146 | CATTGGCTGGTCCCTGATTT | GTTCCCCTATTTGCACATTATTT | 1 | Hom | Het | Het | Het |
| f411 | GCTCCTAATGCGATTTCTCG | CAGCAGGAAGCGTTTAGCTC | 1 | Hom | Het | Het | Het |
| f1177 | GAGAAGATGTGGGATCACTGG | GGTTTCATTAGCAGCCTCCA | 1 | Hom | Hom | Het | Het |
| f536 | CGTGATCGCCCCTGTTAGTA | CAGGAGTGTGGAGAACAGCA | 2 | Het | Het | Het | Het |
| f355 | CCGTTTGGCCCTCTGGTTCTTG | CATCGTACTGCCTCGGGGTTCA | 2 | Het | Het | Het | Het |
| f1733 | CATGGCATAGTGCGAATGTC | TCACGTTGCACCTTCATCAT | 2 | Hom | Het | Het | Het |
| f1734 | GAGACAAGCAGCCTTTCAGC | ATGACTTCCAAGGGCAACAG | 2 | Het | Het | Het | Het |
| f263 | ACTTGCCTGGTGTTTCTCTA | AGCCTTCTGTGATGTCTGC | 2 | Het | Het | Het | Het |
| f1003 | CCTCTGCCTTCTGTCTTTGC | GGATTTCAGGTGTGGGTAGG | 2 | Het | Hom | Het | Het |
| f882 | GCTCCATTTCTCTCTCCATCC | ATAACAGCAGCCGAGGAGAA | 2 | Het | Het | Het | Het |
| f7 | TTAAATGAGGCGAAGGTCGT | ACAGAAAAGCGGCACATTG | 2 | Het | Het | Het | Het |
| f502 | TGCAGGAAGAGTCGGCCATTATCG | CGGGGACGCGGGGACTTTAGA | 2 | Het | Hom | Het | Het |
| f1207 | GCTGATGTTTGTTTGCAGGA | TGTGCACGATTGAGGTTTTC | 2 | Het | Hom | Het | Het |
| f164 | AAGAGTGGCAGGAATGTAATCAAC | ACCTTCCAGCTCTGTCATAGTGC | 3 | Het | Het | Het | Het |
| f137 | ACGGCATCGACACGCATTTTA | CTGGGTTCCTCTCATCTTTGGTTC | 3 | Het | Hom | Het | Het |
| f223 | ACGCATGCCCGCCACAAG | TCCCGCATTAAAATAGAAGAGAA | 3 | Het | Het | Het | Het |
| f121 | TTCCCCTTCTCCCTCTTATTGTTT | AGGCCTGTGAGCACGTGGTAGAGA | 3 | Het | Het | Het | Het |
| f1289 | CAAACCAGGGACAAGTCATAGT | CCCCACTTCCACTATCAACG | 3 | Het | Het | Het | Het |
| f464 | GACGCGTCATGGTCAGTTTA | AGACACCCAGAGCCAGTGAT | 3 | Hom | Het | Het | Het |
| f209 | CCCCTCTTTCCTTCTGTCATCATT | TCTCTGTGGGGTCATCGTGTT | 4 | Het | Hom | Het | Het |
| f76 | GGCCCACTTTTCACACATCT | TCTAAAACTGGCCAGCAGAA | 4 | Het | Het | Het | Het |
| f42 | AGCTGTCTGCCAAAGGAAAA | CCCAGTATCCCAGTGGAGAA | 4 | Het | Hom | Het | Het |
| f607 | CGGTTTTATTTCTGCTGCTTTTT | GTGTGTGTGTGTGTCGGTGA | 4 | Hom | Hom | Het | Het |
| f357 | AGGCTTCACTTTGCTCACG | GGGGGAGGGGGACAACTTTA | 4 | Hom | Hom | Het | Het |
| f207 | TTGCCTCTGTGACGTATGGA | AGATTTGACCCCTCCCTTCC | 4 | Het | Het | Het | Het |
| f1256 | TTGCTTATCGATGGGTTTGA | GTCCAGCTCATGCAAAATCC | 5 | Hom | Het | Het | Het |
| f1294 | AGACGATGATGTGTGCGTGT | CGCATGGACTCCTTTGAAAT | 5 | Het | Het | Het | Het |
| f1252 | CACACCCATAACCACAGCAG | TGGTTCCGATCCATCCATTA | 5 | Het | Het | Het | Het |
| f199 | AGTAGCCTTGTCCTGCCTCATTCA | CTTGGCACGATCTCCCTCCCTCTC | 5 | Het | Hom | Het | Het |
| f1308 | TTTTAGCGTGTGGGGTCTTC | AGGATTTTTCGTCACGGATG | 5 | Het | Het | Het | Het |
| f47 | CACCAGCCAGTCAGTGAGAC | GCCTGAAAATACTGACTGTAGCTG | 5 | Het | Hom | Het | Het |
| f614 | GAGAATATGTCAGAGGGAAGCACT | TAGCGGGTATTGATCAGAAGAGA | 6 | Het | Het | Het | Het |
| f1280 | TAGCCCATCAGGTTCTTTGG | TCTCGCTGACTGCACCTAAA | 6 | Het | Het | Het | Het |
| f416 | GTTGTTGGCACACACTTTGC | GTTAGACTGCGTTTGCGTCA | 6 | Het | Het | Het | Het |
| f112 | GTGACGAGGGGCAACAG | GTGACACCAGACTATACAAAGATG | 6 | Het | Het | Het | Het |
| f1292 | CCACTGTGCTGGAAATGAAA | TCAATGAGACGCCAGCTTTA | 6 | Het | Het | Het | Het |
| f1713 | CAAAACAGCTGTGGCAAAGA | CCAAACAAGCTCAGGGAAAC | 7 | Het | Hom | Het | Het |
| f624 | TCTGCCCTCTACAAGCCTGATAAG | CTCCCTCCTGTCCCACTGCTAATG | 7 | Hom | Het | Het | Het |
| f46 | ATGAGAGCCAGAGACAGGAGGATG | TCAAACAGACGGTCAGCAAAGGAG | 7 | Het | Hom | Het | Het |
| f529 | TTAAACTTTCCCCAGCAGCA | GGCCTAAATGTTCACGTTTCTC | 7 | Hom | Het | Het | Het |
| f327 | GCTGGGCAGACTGATGTGAAT | GAAAGCTCGGGCAAAAAGAAAG | 7 | Het | Het | Het | Het |
| f86 | GTCTCGACTCTCCCTCCCGTTCTG | GGACCGTGACCACATAGCCATACA | 7 | Het | Het | Het | Het |
| f259 | GTGTGATGATGGGATGTTTCTAT | ACTCGGAGCTCTTTTGTGTC | 7 | Hom | Het | Het | Het |
| f367 | CTCCCAATGTAGGTCAGC | CTGCGGAGAGGCGTGATTTGTA | 8 | Het | Het | Het | Het |
| f277 | GAACCGCGACCTGTCTCTAATGG | GCCCGGCTCTGATGTAT | 8 | Hom | Hom | Het | Het |
| f1316 | CCGGGTGGGAAATAAGAGTT | CAGGTACAAAGAGCTGGCAGA | 8 | Het | Hom | Het | Het |
| f63 | CTTGTAGCAGCTGGGAAACGAACC | TCCCCAGGCAAAAAGACGAGACT | 8 | Het | Het | Het | Het |
| f181 | CATGGCCCCAAACACTGAAATA | TCGCGGCCCTCTGAATC | 8 | Het | Hom | Het | Het |
| f396 | CCAACAACGGTCGAAATCATAGAA | CCAGTAATCACCACACGCCAGTA | 9 | Hom | Het | Het | Het |
| f197 | TGGTAAATTAGGCAAGGATTGAA | AAAGCATGCATGTACGCACTA | 9 | Het | Het | Het | Het |
| f256 | ACCCTTCATGGCCTCAGCAGT | GCCCGACCATTAAGCATTTGTT | 9 | Het | Het | Het | Het |
| f62 | TAACCTTAGCCCCATTCT | CAGGCAGTCTCAGCGTGTG | 9 | Hom | Het | Het | Het |
| f1077 | CTGAAAGGGAAAAGCAGCAA | CACGTCAGAAGCTGCGATTA | 9 | Hom | Het | Het | Het |
| f1297 | CCGTCCATGAAAATGGAGAC | GCTGAGCTGGAGAGGAGAAA | 10 | Hom | Hom | Het | Het |
| f471 | GGGTGCATTTCAGTGCTTCT | TCTCTTGCCTCCTTTCTCTCTC | 10 | Het | Het | Het | Het |
| f1298 | CAGGAAGCCAAAGTGCTGTT | GTGCAGGAGGAACCATTGAC | 10 | Het | Hom | Het | Het |
| f1307 | AGTTCGAGCTCCGCAAAGTC | TAGCCCACGTCTAACCAGTG | 10 | Het | Het | Het | Het |
| f1283 | AGTTGCAGAACCTGGAATGG | AGGCTAGAAGAACGCAGCTT | 11 | Het | Het | Het | Het |
| f1014 | AGCCTGCATCACCAGAAACT | AATTATCATGCTGCGCTGTG | 11 | Het | Het | Het | Het |
| f1777 | TGTGGGTTAGGGTCAGAGGT | TCCAACAATCCTTTGGGAGT | 11 | Het | Het | Het | Het |
| f187 | CCTCGAAAGCCCCGGAAGAAGAT | CGGATGATGAGAAATGGGGACTGT | 11 | Het | Het | Het | Het |
| f81 | TCAGGCCCACACGACCACTCA | TCCGCGTTGTTGTTGTTGTTCTTC | 11 | Hom | Het | Het | Het |
| f1804 | CCTCTAGTGGCCGAGCTTTA | CACATTGAAGGTTCGAGCAA | 11 | Het | Het | Het | Het |

| Marker | Foward primer (5'-3') | Reverse primer (5'-3') | Fugu chr | Polymorphisms^a^ | | | |
| --- | --- | --- | --- | --- | --- | --- | --- |
|  |  |  |  | ***T. niphobles*** | ***T. pardalis*** | **F_1_** | **F_1_** |
|  |  |  |  | **granddam** | **grandsire** | **dam** | **sire** |
| f570 | CACGCTGTTTTCACAGATGG | CACAAACACAGATATCGTCTTACCA | 12 | Het | Het | Het | Het |
| f294 | TTAGGGTGAGGGGTGAAAGGTTAG | TATCTCAGTTCAATGGCGTGGTCA | 12 | Het | Het | Het | Het |
| f289 | CTGGAGAAGCTGACGGAGAAATGT | AGTCGAAGCCGTCCAGGTAGGTGA | 12 | Het | Het | Het | Het |
| f125 | AGGACAGCAGAGGAGGAGCAAATG | TCAGATATGGGACAAAGAAGGATG | 12 | Het | Hom | Het | Het |
| f1168 | GAAGCACCACAACACACCAC | CAGGGTTCAGGGAGTTTGAG | 12 | Het | Hom | Het | Het |
| f64 | TGGGGCTGGAGAATGTGTTAGGAT | TCAGCTGTCAGGCGGGGCAAGATG | 12 | Hom | Het | Het | Het |
| f48 | ACTGTAACAAGCATCCGTCCATTC | ACGCCGATCTGCAGTATTAGTTTC | 12 | Het | Het | Het | Het |
| f616 | CATCCGCCCACTGCCTTTTC | TTCAGATATTTTTACCGCTTCACA | 13 | Hom | Het | Het | Het |
| f1274 | CACCAGAGGCTACTGGACAA | AGCCCGGAAAGTATTTCGTT | 13 | Hom | Hom | Het | Het |
| f179 | AAAGTGCCAGGAGTTGTG | TAGAGCTTGTAATGGAAATC | 13 | Het | Hom | Het | Het |
| f1026 | TTCTTGCACAGCCAGAAATG | CGTCTGGTGTGCTTACGTGT | 13 | Het | Het | Het | Het |
| f174 | TTCAGCCAGTAATTATCTATGTA | AAGCCTTGAACTATGAAATGTAAT | 13 | Het | Het | Het | Het |
| f525 | CCTTCAAAGTCGAGCAGAGC | TAACGCAGGCTTGATTTGC | 13 | Het | Het | Het | Het |
| f474 | ACCTCGTCCACATCCTCAAC | ACAAAGCCAGAGGACAATGG | 13 | Het | Het | Het | Het |
| f5 | CCCCACCGCAGCCGACTAAATA | CATCGCCTCTCCAGCAGCAACTT | 14 | Het | Het | Het | Het |
| f448 | GCATTCTGGATGTCAACGTG | ACGGACACACTTCTGTGCTG | 14 | Het | Hom | Het | Het |
| f226 | TCAACATTTCTGAACTGACCTCAT | GCGATCCACAGCAGCATATC | 14 | Het | Het | Het | Het |
| f1278 | TGTGCTGTCAGGCATCTCTT | CCTCCCACAGCACCAGTAAT | 14 | Het | Het | Het | Het |
| f362 | TTACACTGGCCAAACAACTCTG | GGCCTATAGGACCTCTGGACT | 14 | Het | Hom | Het | Het |
| f240 | GGGGGTTGCTGCCTTGTGG | TTTGCGTTTCGGGTTTTTCCTGTT | 14 | Hom | Het | Het | Het |
| f1230 | AAGCCTGAAATGGAGAAGCA | GCACTGCTGTGAGTCTGCTC | 15 | Het | Het | Het | Het |
| f1200 | CCCTGATTCACCCAACTCTG | ACGCACAGCTTGTACCATCA | 15 | Het | Het | Het | Het |
| f469 | GTTGAGCTGAAGGCATCCAT | TGGTTACGCCCAGGTTTATT | 15 | Het | Het | Het | Het |
| f236 | ATCCTGCCTTCAGCAGTGTT | TTGGAGCTGAAAACGAGACA | 15 | Het | Het | Het | Het |
| f617 | CAGGTTGCCCACTATCACTTATTT | CACGGCCGTACTGTCCACT | 16 | Hom | Het | Het | Het |
| f132 | TGCCAGCCCAAAGCGAACAGA | GCTAGCAGGCAGGCAGTAAGG | 16 | Het | Het | Het | Het |
| f1234 | CGAGTGTAGGGCAGCATCTT | CCCATCGTCCTCTTTAGCAC | 16 | Het | Het | Het | Het |
| f1235 | GGTCGTGCACATAGAGCTGA | CTATGCGGTGGTCTCCTAGC | 16 | Het | Hom | Het | Het |
| f102 | CTGCCTGGCTTTTCTTTATCTGTC | AGTTTTACCCGCCTGCTCTTA | 17 | Het | Het | Het | Het |
| f144 | GGGCCACCAATTATCATCAG | TCCAGCTCAGCACCTCCTT | 17 | Het | Het | Het | Het |
| f1581 | CAGTTCAATGCCACCATGAG | TGATGGCTGAATCGTCAGAG | 17 | Hom | Hom | Het | Het |
| f225 | TCCTTCCCTTTCTTTTTCTCTTTA | CTCCCCGGTCGCAGGTA | 17 | Hom | Het | Het | Het |
| f1142 | CTTCTCAGAACGGGTGTGGT | ATCAGCTGCCATGTGATGTC | 18 | Het | Hom | Het | Het |
| f1334 | GCAGCTCTGTGTGTGATCGT | AGGAGGATAAAAGGGCATGG | 18 | Het | Het | Het | Het |
| f204 | GGTACGCTGTTCCACGAG | CACCACTACCATCAACCCCATCTT | 18 | Het | Het | Het | Het |
| f116 | AGCGGGGTGAGAACAAATTA | ATGAGGGAGAGGAGACCACA | 18 | Het | Het | Het | Het |
| f90 | CCTACGACGAGGTGAAGGAG | CAGGTCCTGGTAGGACTGGA | 18 | Hom | Hom | Het | Het |
| f1690 | TCCTCCAACTTCACGCTCTT | AGGTACTTCTGGACCGCATC | 19 | Hom | Het | Het | Het |
| f659 | ACACCTGGTTCAAAGCAACC | GAAATCCTCCAGACCAACCA | 19 | Het | Het | Het | Het |
| f288 | CAAAGAAGGCAGAGAGGTG | TGTCCCGTTCTCTGACACTG | 19 | Het | Hom | Het | Het |
| f70 | GCACTTCTGCAGTGTCATCC | TGCTCCTCTTCACCCTTTTC | 19 | Het | Het | Het | Het |
| f667 | ATGGGACACGGATTGATGAT | GACAACAGGCTTTCAGCACA | 19 | Het | Het | Het | Het |
| f148 | TCTTTGCCAGGAACGACATT | GACGAGGATCAAAGACGGTAAAC | 20 | Hom | Het | Het | Het |
| f128 | AAAGAGCCGCAAACTACTGC | GAGCCGCTCAACAAATGTAA | 20 | Het | Het | Het | Het |
| f252 | GGGCCATAAATACCTCCACAA | GACAACGGCACTATTCTTACTCAA | 20 | Het | Het | Het | Het |
| f1095 | CTAACGGACTGTAGCCAGCA | CCCAACCAACTAACCTCGAA | 20 | Hom | Hom | Het | Het |
| f420 | AGCGTCTCGGTTGAGGTCTA | AAGCTCATTTCAAGGATTTCCA | 20 | Het | Het | Het | Het |
| f1251 | CATGGAAAACGCGTCTCTACT | GTCGCTGCACAGTTCAAAAG | 21 | Hom | Het | Het | Het |
| f1250 | TGCTGCCACCATCATCTTTA | CAGCAGGAGCTCCCATAATC | 21 | Hom | Het | Het | Het |
| f1243 | CCTAGCTTTTCCCACCCATC | ATGCACGGTTCCGTTTTTAG | 21 | Hom | Hom | Het | Het |
| f139 | GCTGCTAACCAGGCTGCTAT | CAAAAGGTTATGCGACATCAGA | 21 | Hom | Het | Het | Het |
| f169 | CTCCCACGCAAGCAGTCA | CTCAGTATCAGGGGTCAAAGAAAT | 21 | Het | Hom | Het | Het |
| f307 | GTTCAGCCCCCTCCACCTACT | CGTCCACGTTCCCAAATCTTAT | 21 | Het | Het | Het | Het |
| f1239 | GACCAACGGGAGCTAAAGTG | GGACTTCACCTCGAAGAACG | 21 | Hom | Het | Het | Het |
| f278 | TGGGAGGGGATTTACAGAGACAC | AAGCAGCGTAGAAACACATCATTG | 22 | Hom | Het | Het | Het |
| f216 | CCCCAAGAGGAGATGAAGAAATG | AGATGGATGGCTAACGGGGAGAG | 22 | Het | Hom | Het | Het |
| f287 | TCGGCGCCACGGGATTA | GTTCTGGCCTCTCACCTGTCACCT | 22 | Het | Hom | Het | Het |
| f1813 | CTGCTCCTGTTTACCCCATC | AGGTGGCCTTAGATGGCATT | 22 | Het | Hom | Het | Het |

**Table S3.** Summary of the linkage map in NP-F_2_ progeny at 104-122 dph

| LG | Number of markers | Length (cM) |
| --- | --- | --- |
| 1 | 7 | 152.3 |
| 2 | 10 | 88.2 |
| 3 | 6 | 94.6 |
| 4 | 6 | 106.3 |
| 5 | 6 | 135.8 |
| 6 | 5 | 75.5 |
| 7 | 7 | 112.1 |
| 8 | 5 | 117.5 |
| 9 | 5 | 107.8 |
| 10 | 4 | 84.5 |
| 11 | 6 | 93.6 |
| 12 | 7 | 76.7 |
| 13 | 7 | 116.6 |
| 14 | 6 | 79.0 |
| 15 | 4 | 86.7 |
| 16 | 4 | 60.7 |
| 17 | 4 | 82.8 |
| 18 | 5 | 88.0 |
| 19 | 5 | 93.8 |
| 20 | 5 | 53.4 |
| 21 | 7 | 116.7 |
| 22 | 4 | 105.7 |
| Total | 125 | 2128.4 |

**Table S4.** Marker position of the linkage map in NP-F_2_ progeny at 104-122 dph

| Marker name | Fugu chr  (NP-F_2_) | Position  (cM) | Marker name | Fugu chr  (NP-F_2_) | Position  (cM) |
| --- | --- | --- | --- | --- | --- |
| f619 | 1 | 0.0 | f81 | 11 | 84.7 |
| f282 | 1 | 24.2 | f1804 | 11 | 93.6 |
| f330 | 1 | 36.6 | f570 | 12 | 0.0 |
| f1712 | 1 | 71.3 | f294 | 12 | 9.3 |
| f146 | 1 | 103.0 | f289 | 12 | 33.1 |
| f411 | 1 | 138.2 | f125 | 12 | 54.2 |
| f1177 | 1 | 152.3 | f1168 | 12 | 57.6 |
| f536 | 2 | 0.0 | f64 | 12 | 62.5 |
| f355 | 2 | 24.1 | f48 | 12 | 76.7 |
| f1734 | 2 | 44.0 | f616 | 13 | 0.0 |
| f1733 | 2 | 44.0 | f1274 | 13 | 12.5 |
| f263 | 2 | 45.3 | f179 | 13 | 17.2 |
| f1003 | 2 | 48.5 | f1026 | 13 | 32.8 |
| f882 | 2 | 49.3 | f174 | 13 | 56.8 |
| f7 | 2 | 58.4 | f525 | 13 | 75.2 |
| f502 | 2 | 75.3 | f474 | 13 | 116.6 |
| f1270 | 2 | 88.2 | f5 | 14 | 0.0 |
| f164 | 3 | 0.0 | f448 | 14 | 22.6 |
| f137 | 3 | 9.4 | f226 | 14 | 30.8 |
| f223 | 3 | 33.2 | f1278 | 14 | 34.5 |
| f121 | 3 | 43.5 | f362 | 14 | 57.6 |
| f1289 | 3 | 64.8 | f240 | 14 | 79.0 |
| f464 | 3 | 94.6 | f1230 | 15 | 0.0 |
| f209 | 4 | 0.0 | f1200 | 15 | 36.2 |
| f76 | 4 | 3.8 | f469 | 15 | 62.8 |
| f42 | 4 | 38.3 | f236 | 15 | 15 |
| f607 | 4 | 52.8 | f617 | 16 | 16 |
| f357 | 4 | 82.9 | f132 | 16 | 16 |
| f207 | 4 | 106.3 | f1234 | 16 | 16 |
| f1256 | 5 | 0.0 | f1235 | 16 | 16 |
| f1294 | 5 | 42.3 | f102 | 17 | 17 |
| f1252 | 5 | 71.3 | f144 | 17 | 17 |
| f199 | 5 | 94.1 | f1581 | 17 | 17 |
| f1308 | 5 | 119.3 | f225 | 17 | 17 |
| f47 | 5 | 135.8 | f1142 | 18 | 18 |
| f614 | 6 | 0.0 | f1334 | 18 | 18 |
| f1280 | 6 | 9.3 | f204 | 18 | 18 |
| f416 | 6 | 33.7 | f116 | 18 | 18 |
| f112 | 6 | 55.5 | f90 | 18 | 18 |
| f1292 | 6 | 75.5 | f1690 | 19 | 19 |
| f1713 | 7 | 0.0 | f659 | 19 | 19 |
| f624 | 7 | 19.8 | f288 | 19 | 19 |
| f46 | 7 | 28.6 | f70 | 19 | 19 |
| f529 | 7 | 49.7 | f667 | 19 | 19 |
| f327 | 7 | 78.0 | f148 | 20 | 20 |
| f86 | 7 | 92.8 | f128 | 20 | 20 |
| f259 | 7 | 112.1 | f252 | 20 | 20 |
| f367 | 8 | 0.0 | f1095 | 20 | 20 |
| f277 | 8 | 34.5 | f420 | 20 | 20 |
| f1316 | 8 | 75.2 | f1251 | 21 | 21 |
| f63 | 8 | 105.9 | f1250 | 21 | 21 |
| f181 | 8 | 117.5 | f1243 | 21 | 21 |
| f396 | 9 | 0.0 | f139 | 21 | 21 |
| f197 | 9 | 25.9 | f169 | 21 | 21 |
| f256 | 9 | 56.0 | f307 | 21 | 21 |
| f62 | 9 | 97.3 | f1239 | 21 | 116.7 |
| f1077 | 9 | 107.8 | f278 | 22 | 0.0 |
| f1297 | 10 | 0.0 | f216 | 22 | 30.7 |
| f471 | 10 | 15.6 | f287 | 22 | 66.8 |
| f1298 | 10 | 47.2 | f1813 | 22 | 105.7 |
| f1307 | 10 | 84.5 | - | - | - |
| f1283 | 11 | 0.0 | - | - | - |
| f1014 | 11 | 9.4 | - | - | - |
| f1777 | 11 | 15.0 | - | - | - |
| f187 | 11 | 47.0 | - | - | - |

**Table S5.** Summary of the linkage map in NS-BC progeny at 42 dph

| LG | Number of markers | Length (cM) |
| --- | --- | --- |
| 1 | 26 | 57.4 |
| 2 | 19 | 40.1 |
| 3 | 11 | 17.3 |
| 4 | 8 | 27.8 |
| 5 | 6 | 20.5 |
| 6 | 11 | 24.4 |
| 7 | 13 | 36.2 |
| 8 | 11 | 9.2 |
| 9 | 12 | 5.8 |
| 10 | 7 | 17.3 |
| 11 | 8 | 30.2 |
| 12 | 10 | 26.8 |
| 13_1 | 10 | 10.1 |
| 13_2 | 2 | 1.1 |
| 14 | 10 | 22.0 |
| 15 | 4 | 24.7 |
| 16 | 13 | 52.8 |
| 17 | 5 | 19.7 |
| 18 | 9 | 54.6 |
| 19 | 16 | 34.1 |
| 20 | 15 | 36.9 |
| 21_1 | 3 | 5.9 |
| 21_2 | 5 | 20.6 |
| 22 | 6 | 26.8 |
| Total | 240 | 622.4 |

**Table S6.** Marker position of the linkage map in NS-BC progeny at 42 dph

| Marker name | Fugu chr  (NS-BC) | Position  (cM) | Marker name | Fugu chr  (NS-BC) | Position  (cM) |
| --- | --- | --- | --- | --- | --- |
| 1313533_60 | 1 | 0.0 | 2583591_107 | 5 | 1.1 |
| 4516789_31 | 1 | 13.1 | 3521802_115 | 5 | 1.1 |
| 5991741_31 | 1 | 13.1 | 7818719_25 | 5 | 14.0 |
| 6334586_30 | 1 | 13.1 | 8362451_77 | 5 | 19.5 |
| 7666039_31 | 1 | 13.1 | 8909218_37 | 5 | 20.5 |
| 8215478_100 | 1 | 13.1 | 133940_35 | 6 | 0.0 |
| 8546301_117 | 1 | 14.2 | 396728_31 | 6 | 0.0 |
| 8960565_48 | 1 | 14.2 | 1057493_24 | 6 | 0.0 |
| 10136070_72 | 1 | 14.2 | 1840714_91 | 6 | 0.0 |
| 10873560_122 | 1 | 14.2 | 3840378_50 | 6 | 0.0 |
| 11768874_70 | 1 | 14.2 | 4330020_82 | 6 | 1.1 |
| 11969347_72 | 1 | 14.2 | 5852721_82 | 6 | 3.5 |
| 14289194_105 | 1 | 18.8 | 6647897_12 | 6 | 4.6 |
| 14590847_57 | 1 | 19.7 | 7890657_89 | 6 | 11.5 |
| 15556430_36 | 1 | 22.8 | 8489770_39 | 6 | 23.2 |
| 17050366_60 | 1 | 26.3 | 8641672_106 | 6 | 24.4 |
| 17872269_44 | 1 | 28.6 | 29371_59 | 7 | 0.0 |
| 18135818_106 | 1 | 28.6 | 1381813_68 | 7 | 0.0 |
| 18790114_52 | 1 | 30.9 | 4309247_76 | 7 | 3.5 |
| 19753772_38 | 1 | 32.6 | 7131439_103 | 7 | 9.2 |
| 20159307_50 | 1 | 33.2 | 7249646_73 | 7 | 11.3 |
| 20177779_73 | 1 | 33.2 | 8039700_47 | 7 | 13.4 |
| 20503064_95 | 1 | 34.3 | 8096666_114 | 7 | 13.4 |
| 21579363_44 | 1 | 45.9 | 9199304_95 | 7 | 13.4 |
| 22734039_84 | 1 | 56.3 | 9325973_70 | 7 | 14.4 |
| 22782272_104 | 1 | 57.4 | 10297429_36 | 7 | 21.2 |
| f794 | 2 | 0.0 | 11634549_50 | 7 | 22.4 |
| 708609_71 | 2 | 0.0 | 12108185_31 | 7 | 27.0 |
| 1250843_69 | 2 | 0.0 | 13111649_37 | 7 | 36.2 |
| 1897244_119 | 2 | 0.0 | 459614_108 | 8 | 0.0 |
| f170 | 2 | 4.4 | 2628420_65 | 8 | 1.1 |
| 3241529_47 | 2 | 8.8 | 4324825_43 | 8 | 2.3 |
| 3433936_55 | 2 | 8.8 | 4821474_12 | 8 | 2.3 |
| f501 | 2 | 11.1 | 4953724_98 | 8 | 2.3 |
| 4084976_37 | 2 | 12.3 | 5128125_96 | 8 | 2.3 |
| 5032003_66 | 2 | 15.7 | 5228698_118 | 8 | 2.3 |
| 5236169_70 | 2 | 15.7 | 5675340_101 | 8 | 3.4 |
| 6488104_75 | 2 | 16.9 | 6957427_113 | 8 | 4.6 |
| f263 | 2 | 16.9 | 7377004_96 | 8 | 5.7 |
| f1844 | 2 | 18.6 | 8563133_106 | 8 | 9.2 |
| f1003 | 2 | 19.2 | 1064104_106 | 9 | 0.0 |
| 7222201_17 | 2 | 19.2 | 1724342_100 | 9 | 1.2 |
| f1833 | 2 | 21.4 | 1957021_51 | 9 | 1.2 |
| f1840 | 2 | 24.7 | 3184710_82 | 9 | 1.2 |
| 10421496_81 | 2 | 40.1 | 3318270_14 | 9 | 1.2 |
| 1003319_64 | 3 | 0.0 | 7478254_79 | 9 | 1.2 |
| 1135614_40 | 3 | 0.0 | 7918633_101 | 9 | 1.2 |
| 1555911_111 | 3 | 0.0 | 9915399_52 | 9 | 2.3 |
| 2803401_35 | 3 | 0.0 | 10607335_82 | 9 | 2.3 |
| 4142176_92 | 3 | 2.3 | 11782311_108 | 9 | 4.6 |
| 5922940_53 | 3 | 6.9 | 11887840_114 | 9 | 4.6 |
| 6407553_67 | 3 | 8.1 | 11912287_13 | 9 | 5.8 |
| 9446111_67 | 3 | 9.2 | 619593_35 | 10 | 0.0 |
| 9627764_27 | 3 | 9.2 | 817085_29 | 10 | 1.2 |
| 10273822_60 | 3 | 11.5 | 3160805_12 | 10 | 1.2 |
| 10947956_53 | 3 | 17.3 | 4038462_68 | 10 | 1.2 |
| 1074728_43 | 4 | 0.0 | 5795293_84 | 10 | 4.6 |
| 3229790_92 | 4 | 0.0 | 6672536_42 | 10 | 13.9 |
| 3764824_14 | 4 | 0.0 | 6863714_105 | 10 | 17.3 |
| 7418628_62 | 4 | 2.3 | 489410_18 | 11 | 0.0 |
| 9623143_86 | 4 | 10.4 | 1671870_73 | 11 | 5.8 |
| 10108609_62 | 4 | 12.6 | 3833715_100 | 11 | 6.9 |
| 11688463_102 | 4 | 25.5 | 7001073_54 | 11 | 14.9 |
| 12096785_14 | 4 | 27.8 | 7227419_57 | 11 | 14.9 |
| 1258491_39 | 5 | 0.0 | 7902927_102 | 11 | 14.9 |

| Marker name | Fugu chr  (NS-BC) | Position  (cM) | Marker name | Fugu chr  (NS-BC) | Position  (cM) |
| --- | --- | --- | --- | --- | --- |
| 9870540_56 | 11 | 20.6 | 860790_26 | 19 | 0.0 |
| 10568362_85 | 11 | 30.2 | 1301242_27 | 19 | 0.0 |
| 122543_11 | 12 | 0.0 | 1645693_57 | 19 | 0.0 |
| 3269813_66 | 12 | 0.0 | 2064671_44 | 19 | 0.0 |
| 3430192_23 | 12 | 0.0 | 2096481_86 | 19 | 0.0 |
| 3468744_25 | 12 | 0.0 | 2230963_111 | 19 | 0.0 |
| 8069333_32 | 12 | 23.4 | 2323001_123 | 19 | 0.0 |
| 8080821_35 | 12 | 24.5 | 2987775_70 | 19 | 0.0 |
| 8235039_28 | 12 | 25.6 | 4775621_65 | 19 | 0.0 |
| 8304875_62 | 12 | 25.6 | 6555705_34 | 19 | 4.6 |
| 8342793_112 | 12 | 25.6 | 7147394_79 | 19 | 5.8 |
| 8837752_99 | 12 | 26.8 | 7451672_16 | 19 | 6.9 |
| 1429041_65 | 13_1 | 0.0 | 7705913_65 | 19 | 6.9 |
| 2742136_19 | 13_1 | 0.0 | 9190916_45 | 19 | 6.9 |
| 2896445_109 | 13_1 | 0.0 | 11639183_71 | 19 | 24.9 |
| 4642361_25 | 13_1 | 0.0 | 12581458_20 | 19 | 34.1 |
| 5294529_30 | 13_1 | 0.0 | 254654_88 | 20 | 0.0 |
| 6666190_19 | 13_1 | 0.0 | 2189297_31 | 20 | 1.7 |
| 8508059_33 | 13_1 | 2.2 | 2784819_48 | 20 | 3.5 |
| 8992336_128 | 13_1 | 5.5 | 4044786_92 | 20 | 4.6 |
| 9859442_54 | 13_1 | 5.5 | 4923471_76 | 20 | 5.8 |
| 13511192_115 | 13_1 | 10.1 | 5149073_13 | 20 | 6.9 |
| 16479637_61 | 13_2 | 0.0 | 6338364_66 | 20 | 10.4 |
| 16670275_81 | 13_2 | 1.1 | 6799155_58 | 20 | 11.5 |
| 332903_69 | 14 | 0.0 | 6940197_72 | 20 | 11.5 |
| 1445757_110 | 14 | 0.0 | 7334192_24 | 20 | 11.5 |
| 3973389_55 | 14 | 2.3 | 10050809_98 | 20 | 17.3 |
| 4046532_25 | 14 | 2.3 | 10962299_54 | 20 | 21.9 |
| 7714536_41 | 14 | 9.2 | 12300005_99 | 20 | 28.8 |
| 8156097_49 | 14 | 9.2 | 13030583_92 | 20 | 34.6 |
| 8673679_107 | 14 | 9.2 | 13324716_73 | 20 | 36.9 |
| 9233921_82 | 14 | 9.2 | 1735367_11 | 21_1 | 0.0 |
| 11497437_15 | 14 | 15.0 | 7405103_92 | 21_1 | 4.8 |
| 12174325_100 | 14 | 22.0 | 8399587_54 | 21_1 | 5.9 |
| 1282791_83 | 15 | 0.0 | 12387134_52 | 21_2 | 0.0 |
| 4091770_16 | 15 | 1.2 | 12870063_57 | 21_2 | 5.8 |
| 7177425_126 | 15 | 10.5 | 13015487_81 | 21_2 | 9.2 |
| 10414363_24 | 15 | 24.7 | 14421470_29 | 21_2 | 14.9 |
| 208719_17 | 16 | 0.0 | 14570844_65 | 21_2 | 20.6 |
| 1278141_89 | 16 | 1.1 | 1976151_60 | 22 | 0.0 |
| 1614533_72 | 16 | 1.1 | 3374070_94 | 22 | 0.0 |
| 2443506_45 | 16 | 6.9 | 3802884_57 | 22 | 0.0 |
| 2741541_74 | 16 | 6.9 | 7917659_91 | 22 | 3.4 |
| 3068024_24 | 16 | 7.8 | 9587226_46 | 22 | 15.1 |
| 4292250_102 | 16 | 12.2 | 10487999_72 | 22 | 26.8 |
| 5075734_39 | 16 | 13.4 | - | - | - |
| 5311354_101 | 16 | 16.5 | - | - | - |
| 5365072_64 | 16 | 17.4 | - | - | - |
| 6172837_78 | 16 | 24.3 | - | - | - |
| 7148736_29 | 16 | 34.8 | - | - | - |
| 10207654_109 | 16 | 52.8 | - | - | - |
| 2215442_122 | 17 | 0.0 | - | - | - |
| 2491165_61 | 17 | 2.3 | - | - | - |
| 2911262_40 | 17 | 8.1 | - | - | - |
| 7120942_19 | 17 | 11.5 | - | - | - |
| 8888232_82 | 17 | 19.7 | - | - | - |
| 1332459_57 | 18 | 0.0 | - | - | - |
| 1998117_117 | 18 | 3.4 | - | - | - |
| 3837118_26 | 18 | 8.0 | - | - | - |
| 4203169_110 | 18 | 8.0 | - | - | - |
| 4753812_89 | 18 | 10.2 | - | - | - |
| 5521705_49 | 18 | 15.9 | - | - | - |
| 6086916_44 | 18 | 27.5 | - | - | - |
| 6941421_15 | 18 | 39.2 | - | - | - |
| 7875186_34 | 18 | 54.6 | - | - | - |

**Table S7.** Summary of phenotypic values

| Cross | QTL mapping | Day post hatch | Sample size | Average ±　S.D. of quantified trait | | |
| --- | --- | --- | --- | --- | --- | --- |
|  |  |  |  | **Number of scales** | **Total area of scales*** | **Average area per scales (Size)*** |
| NP-F_2_ | Genome-wide | 104-122 | 109 | 2.83 ± 2.32 | 560.32 ± 435.09 | 174.96 ± 167.49 |
|  | Chromosome-wide | 149 | 358 | 3.39 ± 2.34 | 595.51 ± 383.34 | 183.80 ± 147.80 |
| NS-BC | Genome-wide | 42 | 85 | Not analyzed | Not analyzed | Not analyzed |
|  | Chromosome-wide | 42 | 203 | Not analyzed | Not analyzed | Not analyzed |
|  | Chromosome-wide | 110 | 222 | Not analyzed | Not analyzed | Not analyzed |
| NS-F_2_ | Chromosome-wide | 109-110 | 196 | 8.25 ± 4.60 | 758.05 ± 500.89 | 93.55 ± 77.35 |

*Pixel unit

**Table S8.** Genotype effects on scale phenotypes in the analysis of binary phenotype. Allele substitution effects at QTL in NP-F_2_ (A) and in NS-BC and NS-F_2_ (B). The allele derived from *T. niphobles*, *T. pardalis* and *T. snyderi* are defined as N, P and S, respectively.

A

| **Cross** | **QTL analysis** | **Day post hatching (dph)** | **Marker** | **Genotype** | **Missing data** | **Phenotype** | |
| --- | --- | --- | --- | --- | --- | --- | --- |
|  |  |  |  |  |  | **Covered** | **Uncovered** |
| NP-F_2_ | Genome-wide | 104-122 | f882 | N/N^a^ | 1 | 21 | 0^b^ |
|  |  |  |  | N/P^a^ |  | 55 | 9 |
|  |  |  |  | P/P |  | 7 | 21 |
|  | Chromosome-wide | 149 | f1821 | N/N^a^ | 0 | 76 | 4 |
|  |  |  |  | N/P^a^ |  | 201 | 2 |
|  |  |  |  | P/P |  | 23 | 52 |

^a^ The phenotypic distributions in N/N and N/P genotypes are not statistically different, while both are significantly different from that in N/N genotype (adjusted *P* < 0.01, Wald test). ^b^ Haldane-Anscombe correction was applied for statistical tests.

B

| **Cross** | **QTL analysis** | **Day post hatching (dph)** | **Marker** | **Genotype** | **Missing data** | **Phenotype** | |
| --- | --- | --- | --- | --- | --- | --- | --- |
|  |  |  |  |  |  | **Covered** | **Uncovered** |
| NS-BC | Genome-wide | 42 | 7222201_17 | N/S^c^ | 0 | 32 | 7 |
|  |  |  |  | S/S |  | 13 | 35 |
|  | Chromosome-wide | 42 | f263 | N/S^c^ | 6 | 71 | 41 |
|  |  |  |  | S/S |  | 9 | 76 |
|  |  | 110 | f1003 | N/S^c^ | 5 | 139 | 13 |
|  |  |  |  | S/S |  | 20 | 45 |
| NS-F_2_ | Chromosome-wide | 109-110 | f1833 | N/N^d^ | 1 | 63 | 2 |
|  |  |  |  | N/P^d^ |  | 99 | 11 |
|  |  |  |  | P/P |  | 7 | 13 |

^c^ The phenotypic distribution in N/S genotype is significantly different from that in S/S genotype (P < 0.01, Wald test). ^d^ The phenotypic distributions in N/N and N/S genotypes are not significantly different, while both are significantly different from that in P/P genotype (adjusted *P* < 1e-04, Wald test).

**Table S9.** List of genes within the 95% credible interval flanking the major QTL on fugu chromosome 2

| No | Annotated gene name | Gene start (bp) | Gene discription |
| --- | --- | --- | --- |
| 1 | LOC101077090 | 6761512 | leucine-rich repeat-containing protein 9-like |
| 2 | rtn1 | 6773218 | reticulon 1 |
| 3 | tdrd9 | 6788608 | tudor domain containing 9 |
| 4 | LOC105418294 | 6801494 | uncharacterized |
| 5 | kif15 | 6802128 | kinesin family member 15 |
| 6 | LOC105418296 | 6806787 | uncharacterized |
| 7 | tacc3 | 6811382 | transforming acidic coiled-coil containing protein 3 |
| 8 | fgfrl1b | 6816759 | fibroblast growth factor receptor-like 1 |
| 9 | zfyve28 | 6838435 | zinc finger FYVE-type containing 28 |
| 10 | cfap99 | 6854306 | cilia and flagella associated protein 99 |
| 11 | LOC105418291 | 6858423 | proprotein convertase subtilisin/kexin type 5-like |
| 12 | mrpl35 | 6862742 | mitochondrial ribosomal protein L35 |
| 13 | reep1 | 6864150 | receptor accessory protein 1 |
| 14 | chmp3 | 6871522 | charged multivesicular body protein 3 |
| 15 | hspa4l | 6875009 | heat shock protein family A (Hsp70) member 4 like |
| 16 | plk4 | 6881106 | polo like kinase 4 |
| 17 | itpk1 | 6886712 | inositol-tetrakisphosphate 1-kinase |
| 18 | LOC101075505 | 6903730 | putative E3 ubiquitin-protein ligase UBR7 |
| 19 | btbd7 | 6910390 | BTB domain containing 7 |
| 20 | unc79 | 6926286 | unc-79 homolog, NALCN channel complex subunit |
| 21 | fam181a | 6967873 | family with sequence similarity 181 member A |
| 22 | LOC101074622 | 6968657 | ankyrin repeat and SOCS box protein 2-like |
| 23 | LOC101074396 | 6977504 | intraflagellar transport protein 140 homolog |
| 24 | otub2 | 6980473 | OTU deubiquitinase, ubiquitin aldehyde binding 2 |
| 25 | ddx24 | 6981738 | DEAD-box helicase 24 |
| 26 | serpina10 | 6985736 | serpin family A member 10 |
| 27 | LOC101073729 | 6987872 | ataxin-3-like |
| 28 | ubr1 | 6992255 | ubiquitin protein ligase E3 component n-recognin 1 |
| 29 | LOC101073279 | 7006598 | photoreceptor outer segment membrane glycoprotein 2-like |
| 30 | tmem179 | 7013823 | transmembrane protein 179 |
| 31 | LOC105418259 | 7015900 | uncharacterized |
| 32 | LOC105418778 | 7022016 | consortin-like |
| 33 | kif26a | 7105570 | kinesin family member 26A |
| 34 | mir203 | 7162438 | microRNA 203 |
| 35 | aspg | 7163715 | asparaginase |
| 36 | LOC101068809 | 7176734 | ADP-ribosylation factor 6 |
| 37 | vcpkmt | 7182721 | valosin containing protein lysine methyltransferase |
| 38 | msh4 | 7185305 | mutS homolog 4 |
| 39 | ktn1 | 7190567 | kinectin 1 |
| 40 | LOC101068358 | 7208391 | E3 ubiquitin-protein ligase pellino homolog 2-like |
| 41 | tmem260 | 7226420 | transmembrane protein 260 |
| 42 | LOC101068129 | 7254991 | homeobox protein OTX2 |
| 43 | LOC105418246 | 7299504 | uncharacterized |
| 44 | exoc5 | 7301136 | exocyst complex component 5 |
| 45 | ap5m1 | 7309722 | adaptor related protein complex 5 subunit mu 1 |
| 46 | slc35f4 | 7316903 | solute carrier family 35 member F4 |
| 47 | LOC101067669 | 7343300 | vascular endothelial growth factor A-like |
| 48 | cipc | 7354064 | CLOCK interacting pacemaker |
| 49 | zdhhc22 | 7359006 | zinc finger DHHC-type containing 22 |
| 50 | tmem63c | 7361261 | transmembrane protein 63C |
| 51 | ngb | 7380790 | neuroglobin |
| 52 | coq6 | 7389457 | coenzyme Q6, monooxygenase |
| 53 | entpd5 | 7397795 | ectonucleoside triphosphate diphosphohydrolase 5 |
| 54 | bbof1 | 7406325 | basal body orientation factor 1 |
| 55 | aldh6a1 | 7411829 | aldehyde dehydrogenase 6 family member A1 |
| 56 | gstz1 | 7417369 | glutathione S-transferase zeta 1 |
| 57 | LOC101069658 | 7419144 | acyl-CoA-binding domain-containing protein 5-like |
| 58 | tmed8 | 7423108 | transmembrane p24 trafficking protein family member 8 |
| 59 | atg2b | 7426442 | autophagy related 2B |
| 60 | gskip | 7441000 | GSK3B interacting protein |
| 61 | npc2 | 7443442 | NPC intracellular cholesterol transporter 2 |
| 62 | isca2 | 7445589 | iron-sulfur cluster assembly 2 |
| 63 | eml5 | 7448203 | echinoderm microtubule associated protein like 5 |
| 64 | ttc8 | 7472237 | tetratricopeptide repeat domain 8 |
| 65 | LOC101068741 | 7476239 | CD209 antigen-like |

| No | Annotated gene name | Gene start (bp) | Gene discription |
| --- | --- | --- | --- |
| 65 | LOC101068741 | 7476239 | CD209 antigen-like |
| 66 | LOC105418215 | 7480983 | CD209 antigen-like protein E |
| 67 | LOC105418214 | 7483941 | CD209 antigen-like protein C |
| 68 | LOC105418212 | 7489402 | CD209 antigen-like protein C |
| 69 | nek9 | 7497667 | NIMA related kinase 9 |

**Table S10.** The relationship between scale phenotypic and habitat in *Takifugu* species

| Phenotype | Species | Habitat | Spawing | Ref. |
| --- | --- | --- | --- | --- |
| Scale-uncovered phenotype | *T. porphyreus* | sea | sea | 1, 2 |
|  | *T. vermicularis* | sea, demersal | sea | 3 |
|  | *T. chrysops* | sea, demersal | sea | 3 |
|  | *T. pardalis* | sea, river mouth | sea | 1, 2 |
|  | *T. snyderi* | sea, demersal | sea | 3 |
| Scale-covered phenotype | *T. oblongus* | sea, brackish, demersal | sea | 3 |
|  | *T. obscurus* | sea, river | river | 2, 4, 5, 6, 7 |
|  | *T. rubripes* | sea, river mouth | sea | 1, 2, 4 |
|  | *T. poecilonotus* | sea, river mouth | sea | 2, 4 |
|  | *T. exascurus* | sea, demersal | sea | 8 |
|  | *T. xanthopterus* | sea, river mouth, brackish | sea | 3, 8 |
|  | *T. ocellatus* | sea, river, brackish, demersal | river | 8 |
|  | *T. niphobles* | sea, river mouth | sea | 2, 4  / |
|  | *T. stictonotus* | sea, river mouth, demersal | sea | 8 |

**Reference for supplementary Tables**

1. Fujita, S. Studies on life history and aquaculture of japanese puffer fishes. *Rep. Nagasaki. Pref. Inst. Fish* **1962**, *2*, 1–121.
2. Masuda, H.; Amaoka, K.; Araga, C.; Uyeno, T.; Yoshino, T. *The Fishes of the Japanese Archipelago*, *(Plates)*; Tokai University Press: Shinjuku Tokai Building, Tokyo, Japan 1984.
3. ICUN Red List. Available online: http://www.iucnredlist.org/ (accessed on 12 October 2019).
4. Kim, I.S. Illustrated encyclopedia of fauna and flora of Korea. In *Freshwater Fishes*; Ministry of Education: Seoul, Korea, 1997; Volume 37, p. 629.
5. Miyadi, D.; Kawanabe, H.; Mizuno, N. *Coloured Illustrations of the Freshwater Fishes of Japan*; Hoikusha: Osaka, Japan, 1976.
6. Ni, Y.; Wang, Y.; Xu, C.; Xiong, G. *The Fishes of Shanghai Area*; Science and Technology Press in Shanghai: Shanghai, China, 1990.
7. Abe, T. Taxonomic studies on the puffers (Tetraodontidae, Teleostei) from Japan and adjacent Regions-V. synopsis of the puffers from Japan and adjacent regions. *Bull. Biogeogr. Soc. Japan* **1949**, *14*, 89–140.
8. Fishbase. Available online: http://www.fishbase.org/ (accessed on 12 October 2019).
