# Supplementary material for: The Genetic Basis of Scale-Loss Phenotype in the Rapid Radiation of Takifugu Fishes
Source: Genes (Basel). 2019 Dec 10;10(12):1027. doi: 10.3390/genes10121027 (PMC6947334; doi:10.3390/genes10121027)
Supplement: Supplementary file 1 [file genes-10-01027-s001.zip › supplementary files revised191206/SuppleText.docx]

**1. Genetic mapping**

library(qtl)

setwd("C:/Users/KDI/Desktop/mapping")

mapping <- read.cross("csv",

file="20181102_Linkagemapping_NS_BC_KD100_finalver.csv", estimate.map = FALSE)

summary(mapping)

plotMissing(mapping)

par(mfrow=c(1,2),las=1)

plot(ntyped(mapping), ylab="No. typed

markers", main="No. genotypes by individual")

plot(ntyped(mapping, "mar"),

ylab="No. typed individuals", main="No. genetypes by marker")

mapping <- subset(mapping,

ind=(ntyped(mapping)>265))

nt.bymar <- ntyped(mapping,"mar")

mapping <- subset(mapping, ind=(ntyped(mapping)>230))

nt.bymar <- ntyped(mapping, "mar")

todrop <- names(nt.bymar[nt.bymar<80])

cg <- comparegeno(mapping)

hist(cg[lower.tri(cg)], breaks=seq(0, 1,

len=101), xlab="No. matching genotypes")

rug(cg[lower.tri(cg)])

wh <- which(cg > 0.9, arr=TRUE)

wh <- wh[wh[,1] < wh[,2],]

wh

gt <- geno.table(mapping)

gt[gt$P.value < 0.05/totmar(mapping),]

todrop <- rownames(gt[gt$P.value < 1e-10,])

mapping <- drop.markers(mapping, todrop)

g <- pull.geno(mapping)

gfreq <- apply(g, 1, function(a)

table(factor(a, levels=1:2)))

gfreq <- t(t(gfreq) / colSums(gfreq))

par(mfrow=c(1,2), las=1)

for(i in 1:2)

plot(gfreq[i,], ylab="Genotype frequency", main=c("AA","AB")[i], ylim=c(0,1))

mapping <- est.rf(mapping)

checkAlleles(mapping, threshold=3)

rf <- pull.rf(mapping)

lod <- pull.rf(mapping, what="lod")

plot(as.numeric(rf), as.numeric(lod),

xlab="Recombination fraction", ylab="LOD score")

lg <- formLinkageGroups(mapping, max.rf=0.4, min.lod=4)

table(lg[,2])

mapping <- formLinkageGroups(mapping,

max.rf=0.4, min.lod=4, reorgMarkers = TRUE)

plotRF(mapping, alternate.chrid=TRUE)

est <- est.map(mapping, map.function = "kosambi")

pull.map(mapping)

mapping <- replace.map(mapping, est)

pull.map(mapping)

mycross <- replace.map(mycross, mymap)

mymapAsTable <- pull.map(mymap, as.table=TRUE)

write.csv(mymapAsTable, file="20181102mymap.csv")

**2. QTL analysis**

library(qtl)

wd <- "D:/Desktop/Spine_QTL/Data/Kim_BC_42/data for qtl_Kim_BC_42_new_20181020"

file1 <- "20181102_Linkagemapping_NS_BCcollected_KD1.csv"

setwd(wd)

data <- read.cross(file=file1, "csv", estimate.map=FALSE)

est <- est.map(data, map.function="kosambi")

map <- replace.map(data, est)

error <- calc.errorlod(map, error.prob=0.01)

spine <- calc.genoprob(error, step=1, error.prob=0.01)

cg <- calc.genoprob(map, step=1)

one <- scanone(cg, pheno.col=1, model="binary", method="em")

perm <- scanone(cg, pheno.col=1, method="em", model="binary", n.perm=10000, n.cluster=4)

plot(one, show.marker.names=FALSE)

add.threshold(one, perms=perm, alpha=0.01, col="black", lty=2, lwd=2)

add.threshold(one, perms=perm, alpha=0.05, col="black", lty=2, lwd=2)

add.threshold(one, perms=perm, alpha=0.001, col="black", lty=2, lwd=2)

bayesint(one, chr=2, prob=0.95, expandtomarkers=TRUE)

segments(11.77942,1.20,36.84796,1.20, lwd=2, col="black")

write.table(bi, "95perCI_BC_42_Binary_renewal20180509.csv", sep=",", quote=FALSE)

 (bi <- bayesint(one, chr3, prob=0.95, expandtomarkers=TRUE))

write.table(bi, "95perCI_BC_42_Binary_renewal20180510.csv", sep=",", quote=FALSE)

 find.marker(map, chr=2, pos=20.0)

qc1 <- c("2")

qp1 <- c(20.0)

 spinesubset1 <- subset(spine, chr=qc1)

spine.a1 <- sim.geno(spinesubset1, n.draws=128, err=0.001)

qtl1 <- makeqtl(spine.a1, qc1, qp1)

 summary(qtl1)

lod1 <- fitqtl(spine.a1, pheno.col=1, qtl1, formula=y~Q1, method="imp", model=c("binary"), dropone=TRUE, get.ests=TRUE)

summary(lod1)

mar <- find.marker(map, chr=2, pos=20.0)

plotPXG(map, pheno.col=1, marker=mar)

eff <- effectplot(map, mname1 = "Chr2_7222201_17")

eff

options(max.print=1000000)

one

data <- read.table("clipboard", header = T)

 head(data)

kruskal.test(data$Pheno~data$Geno)

install.packages("PMCMR")

library("PMCMR", lib.loc="~/R/win-library/3.3")

set.seed(124)

data$Rank <- rank(data$Pheno, ties.method="random")

posthoc.kruskal.dunn.test(x=data$Rank, g=data$Geno, method="bonferroni")

posthoc.kruskal.dunn.test(x=data$Rank, g=data$Geno, method="fdr")
